# Spatiotemporal optimization of micro-spray misting for orchard microclimate regulation and water-use efficiency in semi-arid apple orchards

**DOI:** 10.3389/fpls.2026.1895726

**Published:** 2026-08-18

**Authors:** Hanmi Zhou, Sibo Lu, Yumin Su, Jichen Li, Jiankun Ge, Xinguang Wei, Youzhen Xiang, Qi Wu, Linshuang Ma, Cheng Chen, Zhe Peng, Ru Huang

**Affiliations:** 1 College of Agricultural Equipment Engineering, Henan University of Science and Technology, Luoyang, Henan, China; 2 School of Water Conservancy, North China University of Water Resources and Electric Power, Henan Key Laboratory of Crop Water Use, Zhengzhou, China; 3 College of Water Conservancy, Shenyang Agricultural University, Shenyang, Liaoning, China; 4 Key Laboratory of Agricultural Soil and Water Engineering in Arid and Semi-arid Areas, Ministry of Education, Northwest A&F University, Yangling, Shaanxi, China

**Keywords:** apple, canopy cooling, canopy microclimate, eco-physiological response, heat and drought stress, micro-spray misting, photosynthesis, water-use efficiency

## Abstract

Global warming has intensified heat and drought stress in semi-arid orchard systems, posing major challenges to sustainable apple production. Although micro-spray misting can simultaneously regulate canopy cooling and humidification, orchard responses to different spatiotemporal misting configurations remain insufficiently understood. In this two-year field study, we evaluated the effects of three misting heights (upper, middle, and lower canopy) and four misting durations (0.5–2.0 h) on orchard microclimate, physiological activity, fruit production, and water-use performance in semi-arid apple (*Malus domestica* Borkh.) orchards. Moderate misting applied within the middle canopy for 1.5 h (H2T2) most effectively improved the orchard microenvironment, reducing average canopy temperature by 4.3 °C and increasing relative humidity by 5.7%. This treatment enhanced photosynthetic performance and water-use efficiency at both leaf and orchard scales and resulted in the highest fruit yield and best overall fruit quality. Comprehensive multi-criteria evaluation further placed the 1.0 h middle-canopy treatment (H2T3) in the same top-performance tier as H2T2. Moving beyond discrete treatment-level comparisons, response surface analysis identified a continuous range of near-optimal parameter combinations within the tested parameter space. Specifically, a duration of 0.70–1.46 h and a misting height of 74–113 cm maintained ≥95% of the maximum predicted response, revealing a stable and feasible operational range rather than a single optimum under field conditions. These findings demonstrate that orchard responses to micro-spray misting are governed by both misting height and operating duration, and that moderate middle-canopy misting with intermediate operating duration provides a practical management strategy for optimizing canopy microenvironments and water-use efficiency under heat and drought stress in semi-arid apple production systems.

## 1 Introduction

As global warming intensifies, the increasing frequency of extreme drought and heat events has become a major constraint on agricultural production, particularly in semi-arid regions (Kumar et al., 2023; Saleem et al., 2025). In orchard systems, water scarcity combined with high evaporative demand poses a critical challenge because both productivity and resource-use efficiency must be maintained under limited irrigation conditions. Apple is one of the most widely cultivated temperate fruit crops and is predominantly grown in the semi-arid northern regions of China, where water limitation has become a key bottleneck restricting high-quality and high-yield production (Ning et al., 2024; Shi et al., 2025). Drip irrigation improves water-use efficiency mainly by replenishing root-zone water. However, it cannot directly regulate canopy temperature and humidity under hot and dry conditions, which limits its capacity to alleviate transpiration stress and heat-induced physiological injury (Devin et al., 2023). Previous studies have shown that canopy microclimate, especially temperature and humidity, strongly influences leaf photosynthetic activity, transpiration, and tree stress tolerance (Reta et al., 2025; Zhen et al., 2025). In apple orchards, canopy structure and vertical heterogeneity can further modify the spatial distribution of temperature and humidity within the canopy, thereby affecting physiological performance and fruit development (Blanco and Kalcsits, 2024; Herpin et al., 2024; Xu et al., 2024). These findings suggest that effective orchard adaptation to heat and drought requires irrigation strategies capable of simultaneously regulating soil water supply and canopy microenvironment.

Micro-spray misting, as a form of micro-spray irrigation, offers such a possibility by regulating canopy temperature and humidity while also supplementing local moisture to some extent (Zheng et al., 2021; Xue et al., 2024). Compared with drip irrigation alone, micro-spray misting may enhance evaporative cooling and canopy humidification, whereas compared with conventional sprinkler irrigation, it generally results in lower evaporation losses and higher water-use efficiency. Previous studies have shown that micro-spray misting can alleviate drought stress, improve photosynthetic performance, and enhance crop yield and quality (Liu et al., 2020; Xue et al., 2023). Xue et al. (2025) confirmed that micro-spray misting effectively improved tomato growth and fruit quality, restored the photosynthetic mechanism disrupted by high temperature, and reduced the photoprotective mechanism, actively dissipating excess energy as heat. Zheng et al. (2022) found that the combination of canopy micro-spray and shading can effectively regulate the canopy microclimate of drip-irrigated vineyards and create favorable conditions for maintaining stable physiological activity of leaves. However, most existing studies have focused primarily on individual operational factors such as water volume or spray frequency, while the combined effects of canopy position (spatial scale) and misting duration (temporal scale) remain insufficiently understood. In apple orchards, canopy height may influence droplet interception patterns and the spatial uniformity of evaporative cooling within the canopy, whereas misting duration may influence humidity accumulation and canopy transpiration demand under prolonged heat exposure. Consequently, these spatiotemporal factors are likely to jointly affect canopy cooling efficiency, physiological activity, and orchard water-use performance. Although micro-spray misting has shown promise in vegetable (Xue et al., 2025) and vineyard systems (Zheng et al., 2022), and recent studies have confirmed that overhead evaporative cooling can significantly improve fruit tree canopy microclimate and productivity under heatwave conditions (Dag et al., 2026), spatiotemporal optimization of misting configurations—particularly the combined effects of canopy position and misting duration—remains largely unexplored in pome fruit orchards, where perennial canopy structure and vertical heterogeneity introduce additional complexity (Chandel et al., 2025; Rodrigues et al., 2025).

Because micro-spray misting simultaneously influences canopy microclimate, physiological activity, fruit production, and water consumption, evaluating individual variables alone may not fully reflect the coordinated responses and trade-offs among microclimate regulation, productivity, and water-use efficiency (He et al., 2021b; Hao et al., 2022; Cai et al., 2023). In practical orchard management, however, operational parameters rarely remain at fixed discrete levels, and small fluctuations in misting height or duration may influence the balance among canopy cooling, physiological activity, and water use. Therefore, identifying stable and feasible operational ranges may be more informative than relying solely on single-treatment optima. Accordingly, this two-year field study systematically investigated the spatiotemporal regulation effects of micro-spray misting in apple orchards under semi-arid conditions, with particular emphasis on identifying practically applicable misting conditions. The study aimed to: (1) quantify the effects of misting height and duration on orchard microclimate, physiological activity, and water-use efficiency; (2) evaluate the associated impacts on fruit yield and quality; and (3) identify stable operational ranges capable of maintaining favorable orchard responses under heat and drought stress. The results provide practical support for improving orchard microclimate regulation and misting management under increasing heat and drought stress in semi-arid orchard systems.

## 2 Materials and methods

### 2.1 Overview of the experimental site

**Figure 1:**
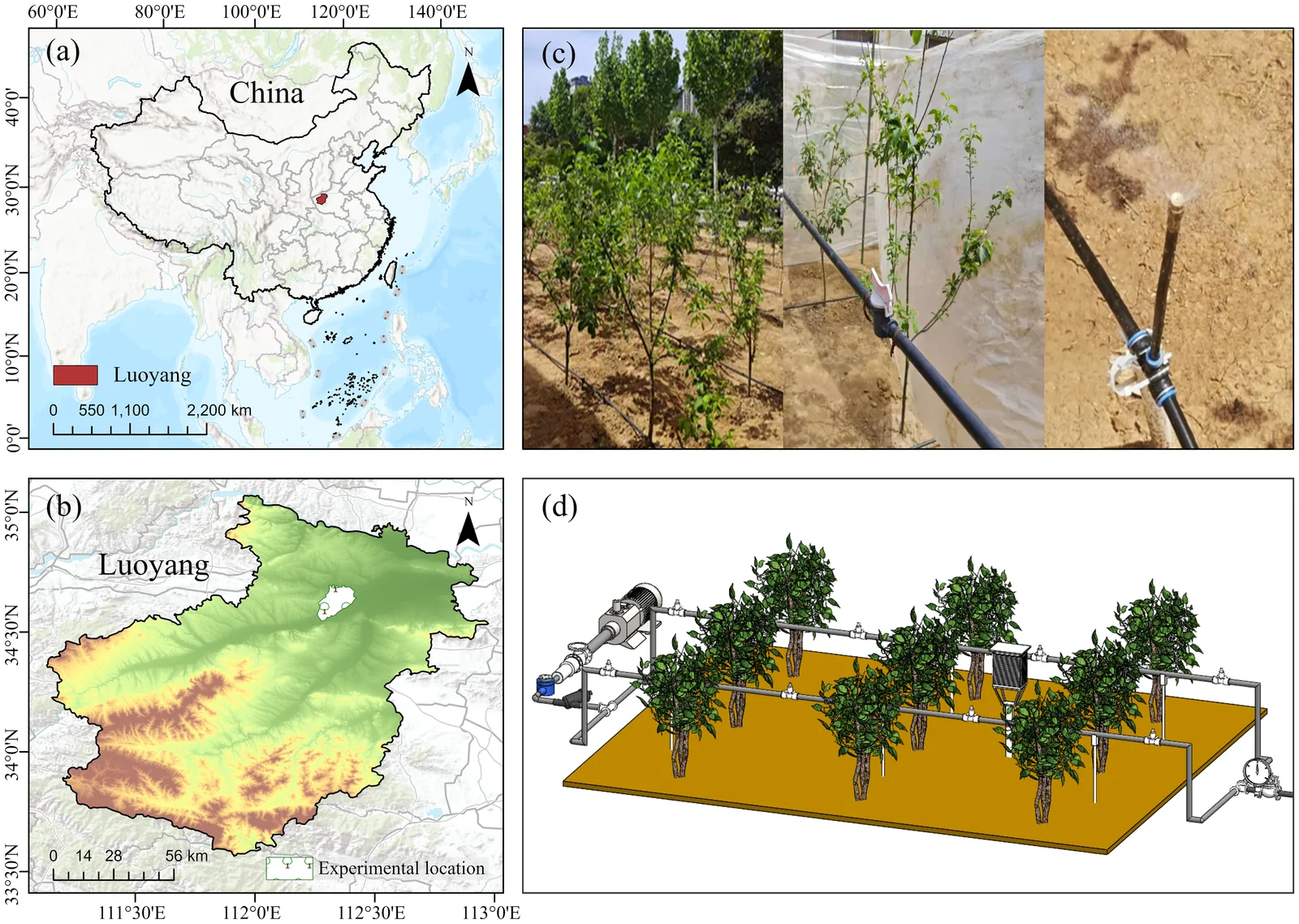
Overview of the experimental site, showing the geographic location of the study area in China **(a)**, topography of the Luoyang region **(b)**, field view of the orchard and micro-spray irrigation system **(c)**, and schematic of the irrigation system **(d)**.

The experiment was conducted in 2024 and 2025 at the Water-Saving Irrigation Experimental Station of Henan University of Science and Technology in Luoyang City, Henan Province, China (34° 40′ N, 112° 22′ E). The site represents a typical northern semi-arid region, with an elevation of 172 m (**Figure 1**). The annual average temperature ranges from 12.2 to 24.6 °C, with a minimum of 6 °C and a maximum of 44 °C during the trial period. Annual precipitation averages approximately 600 mm, while annual evaporation reaches 1200 mm. The frost-free period exceeds 210 days, and annual sunshine hours range from 2200 to 2300. The soil at the experimental site was classified as cinnamon soil, with a field water-holding capacity (θ*_f_*) of 21.3%, a bulk density of 1.1 g cm^−3^, and a pH of 7.8. Soil ammonium nitrogen, nitrate nitrogen, available phosphorus, and available potassium contents were 9.47, 4.99, 35.10, and 239.14 mg kg^−1^, respectively. The test material was the ‘Yanfu No. 8’ apple cultivar (grafted onto *Pingyi* Sweet Tea rootstock). The selected apple trees were comparable in size and canopy shape.

### 2.2 Experimental design

The experiment followed a 3 × 4 full factorial design with four replications per treatment, comprising 12 strictly isolated plot units. Tree spacing was 1.6 × 1.6 m, and each treatment was established as a square plot. A double-layer waterproof membrane was buried to a depth of 1.5 m between plots to prevent lateral seepage, and a 2.4-m-high plastic film barrier was installed between adjacent treatments to minimize mist drift. The experimental orchard adopted a combination of drip irrigation and micro-spray misting. Drip irrigation was used to supplement soil moisture, with two emitters installed beneath each tree at a distance of 0.10 m from the trunk to ensure a uniform water supply within the root zone. Irrigation was initiated when soil moisture content declined to the preset lower threshold of 65% θ*_f_* and was terminated once it reached the upper threshold of 80% θ*_f_*. The amount of water applied during each irrigation event was calculated according to the field water-holding capacity and precisely controlled using a water meter to ensure adequate soil moisture for tree growth.

Micro-spray misting was applied as the experimental treatment to regulate canopy microclimate under sunny conditions. The experiment included two factors: misting duration and misting height. Misting duration had four levels, namely 2.0 h (full misting, T1), 1.5 h (moderate misting, T2), 1.0 h (light misting, T3), and 0.5 h (minimal misting, T4), all initiated at 11:00 a.m. Misting height was set at three levels according to tree height and canopy distribution: 1.5 m above ground (upper canopy, H1), 1.0 m (middle canopy, H2), and 0.5 m (lower canopy, H3). The micro-spray system had a spray radius of 1.5 m and a flow rate of 12 L h^−1^. During the experimental period, micro-spray misting was conducted nine times in 2024 and thirteen times in 2025 according to weather conditions.

The three misting heights (0.5, 1.0, and 1.5 m above the ground) were selected to correspond to the lower, middle, and upper canopy layers of apple trees, thereby capturing the vertical heterogeneity in canopy architecture, solar radiation interception, and local microclimatic conditions. The four misting durations (0.5, 1.0, 1.5, and 2.0 h) were selected to represent a gradient from minimal misting to prolonged daytime cooling during periods of high solar radiation and atmospheric evaporative demand, thereby enabling the evaluation of both insufficient and excessive misting effects on canopy microclimate regulation. Misting was initiated at 11:00 a.m. under sunny conditions. Based on our previous research on orchard misting management, this time was selected as the standardized misting initiation time. Under local summer conditions, this period generally corresponds to the stage when solar radiation and atmospheric evaporative demand begin to increase rapidly. To isolate the effects of misting height and duration, the initiation time was kept constant across all treatments.

To ensure consistency among treatments, all orchard management practices were kept uniform throughout the experimental period, including annual pruning, flower and fruit thinning, and integrated water–fertilizer management. One basal fertilizer application and two top-dressing applications were applied during each growing season. The total application rates during the growing season were 432 kg N ha^−1^, 288 kg P_2_O_5_ ha^−1^, and 240 kg K_2_O ha^−1^. Based on local orchard production experience and the morphological and physiological characteristics of apple trees, the growth period was divided into five stages: bud break and flowering, shoot growth, fruit setting, fruit enlargement, and fruit maturity (**Table 1**). Among these, the bud break and flowering, fruit setting, and fruit enlargement stages were identified as the critical growth stages because of their central roles in physiological activity, fruit development, and yield formation. All measurements and observations were conducted according to this growth-stage classification.

**Table 1:** Growth stage division of apple trees in 2024 and 2025.

Growth stage	2024	2025
Bud break and flowering	Mar 30 – Apr 28	Mar 27 – Apr 26
Shoot growth	Apr 29 – May 28	Apr 27 – May 27
Fruit setting	May 29 – Jun 27	May 28 – Jun 27
Fruit enlargement	Jun 28 – Jul 27	Jun 28 – Jul 28
Fruit maturity	Jul 28 – Aug 26	Jul 28 – Aug 27

### 2.3 Observation indicators and methods

#### 2.3.1 Growth increment

Basal stem growth (mm): A measurement mark was made at the base of the apple tree rootstock. The diameter was measured in cross-section using a digital vernier caliper. Measurements are taken once at the beginning and once at the end of each growth stage. The difference in diameter between the two measurements represents the basal stem growth of the apple tree during that growth stage.

Plant growth (cm): The vertical height from the ground to the highest point of the apple tree was measured using a tape measure. Each growth stage was measured once, and the difference between the two measurements was the plant’s growth during that period.

Leaf area (LA, m²): The leaf area of a single leaf was measured using a LI-3000C handheld leaf area meter (Li-Cor, USA) once in each growth stage during the apple growing season. Ten leaves were randomly selected from the upper, middle, and lower directions, and the average value was taken as the single leaf area. Leaf area per plant was estimated using a non-destructive approach: individual leaf area was measured with a portable leaf area meter on a representative subsample, and total leaf number per plant was estimated by counting all leaves on three representative branches per tree and scaling proportionally to the total branch count.

#### 2.3.2 Relative chlorophyll content

Relative chlorophyll content (SPAD) of apple leaves was measured using a SPAD-502 portable chlorophyll meter (Konica Minolta, Japan). For each plant, five leaves were collected from each of the upper, middle, and lower canopy layers, and the measurements were averaged. The measurement was taken at a position two-thirds of the leaf blade length from the petiole. Three plants per treatment were measured, and the mean value was used for analysis. Measurements were conducted once at each growth stage.

#### 2.3.3 Photosynthetic characteristics

Photosynthetic rate (Pn, μmol CO_2_·m^−^²·s^−1^) and transpiration rate (Tr, mmol H_2_O·m^−^²·s^−1^) were measured using an SC-3051D portable photosynthesis meter (Top Cloud Agriculture, China). Measurements were taken on intact upper-middle leaves of apple trees under different treatments. Sampling was conducted on sunny mornings at 10:00 am during each growth stage. For each apple tree, five leaves were selected and marked to ensure the same leaves were measured across all sampling times. Three stable readings were recorded per leaf, and the mean of these stable values was used as the final value for each treatment. Leaf instantaneous water use efficiency (LWUE, μmol CO_2_ (mmol H_2_O)^−1^) was calculated as Pn/Tr. Measurements were performed once per growth stage.

#### 2.3.4 Chlorophyll fluorescence parameters

Chlorophyll fluorescence parameters were measured using a MINI-PAM-II portable modulated chlorophyll fluorometer (WALZ, Germany). Measurements were taken on intact upper-middle leaves of apple trees under different treatments. For each apple tree, three leaves were selected and marked. Maximum photochemical quantum yield of photosystem II (PSII) (Fv/Fm): The marked leaf area was dark-adapted for 20 min using a dark adaptation clip. Measurements were performed with actinic light turned off. Light-adapted parameters: Effective quantum yield of PSII [Y(II)] and photosynthetic electron transport rate (ETR) were determined under actinic red light (peak wavelength ~ 635 nm) at an intensity of 45 μmol photons m^−^² s^−1^. Three trees per treatment were measured, and the mean values were used for analysis. Measurements were conducted once at each of three key growth stages.

#### 2.3.5 Fruit yield and quality assessment

Because the experimental trees were still in the juvenile vegetative phase in 2024 and did not bear fruit, fruit yield and quality were evaluated only at maturity in 2025. All fruits from each tree were harvested and weighed using an electronic balance to determine the yield per tree (g tree^−1^). Fruit hardness (kgf cm^−^²) was measured using a GY-4 fruit hardness tester (Topyun Nong, China). Three measurement points were selected on the equatorial region of each fruit. After removing the peel, flesh firmness was measured at each point, and the average value was recorded. Soluble solids content (°Brix) was determined using a PAL-1 portable digital refractometer (ATAGO, Japan). Three replicate measurements were taken per fruit, and the mean value was calculated for each treatment.

#### 2.3.6 Canopy temperature and humidity measurement

On clear days during critical growth stages, canopy temperature and humidity were measured using an AS817 handheld thermo-hygrometer (Sima, China). Measurements were taken immediately after micro-sprinkler misting treatment. At each misting location, temperature and humidity were recorded at the mid-canopy level of three apple trees. Ambient temperature and humidity were measured simultaneously. The absolute differences between canopy and ambient temperature and humidity were then calculated.

#### 2.3.7 Water use efficiency and fertilizer partial productivity calculation

Water use efficiency (WUE, kg·m^−3^) was calculated using Equation 1:

(1) $$\text{WUE}=\frac{\text{Y}}{\text{ET}}$$

where *Y* is the yield (kg), and *ET* is the crop water consumption (m^3^).

Crop water consumption was calculated according to the water balance method (Zhong et al., 2019), as expressed in Equation 2.

(2) $$\text{ET}=P_{r}+U+I-D-R-\Delta W$$

Where *Pr* is precipitation, *U* is groundwater recharge, *I* is irrigation volume, *D* is deep percolation, *R* is runoff, and Δ*W* is the change in soil water storage between the beginning and end of the experiment (Li et al., 2024).

In this study, multiple terms in the full water balance equation were assumed negligible based on the experimental design and local hydrogeological conditions. Double-layer anti-seepage plastic membranes were buried to a depth of 1.5 m around each plot to minimize lateral water exchange among plots. Surface runoff was considered negligible because irrigation was precisely controlled and no surface water accumulation occurred during the experiment. According to the Luoyang Water Resources Bulletin 2021 (Luoyang Water Resources Bureau, 2022), groundwater tables in the central urban area of Luoyang are substantially deeper than the effective rooting zone of apple trees. Therefore, groundwater contribution through upward capillary flux was considered negligible under the hydrogeological conditions of the present study. Although limited capillary rise may occur in shallow groundwater systems during prolonged drought periods, such conditions do not apply to the present experimental site. Soil moisture was maintained within 65–80% of field water-holding capacity through controlled irrigation, thereby reducing the probability of substantial deep percolation below the root zone. In addition, changes in soil water storage between the beginning and end of the growing season were determined from soil moisture measurements obtained using the soil sampling and oven-drying method and represented only a small proportion of the total seasonal water input (precipitation plus irrigation). Therefore, *U*, *R*, *D*, and *ΔW* were considered negligible under the experimental conditions, and *ET* was estimated according to Equation 3.

(3) $$\text{ET}=P_{r}+I$$

Partial factor productivity (*PFP*, kg·kg^−1^) was calculated using Equation 4:

(4) $$\text{PFP}=\frac{\text{Y}}{{\text{F}}_{\text{T}}}$$

where *F_T_* is the total amount of applied fertilizer (N + P_2_O_5_ + K_2_O, kg).

### 2.4 Comprehensive evaluation

The Analytic Hierarchy Process (AHP) is a subjective weighting method. Its principle is to systematically decompose complex decision-making problems into a hierarchical structure model based on expert experience and judgment. Elements such as objectives, criteria, and alternatives are layered according to their hierarchical relationships. The relative importance of each element is quantified through pairwise comparison matrices, and the consistency test ensures logical rationality (Veisi et al., 2022). AHP emphasizes the combination of qualitative analysis and quantitative calculation, effectively capturing the preferences and perceptions of decision-makers. It is suitable for multi-criteria decision-making problems in selecting the best irrigation systems (Veisi et al., 2022).

The Coefficient of Variation (CV) method is an objective weighting method. Its principle is to use the CV of data to measure the relative volatility of indicators. The larger the CV, the more significant the differences among different evaluation objects, and the richer the information content it carries (Taherdoost and Mohebi, 2024). This method is entirely based on the statistical properties of the data itself, without human intervention, and can objectively reflect the intrinsic differences among indicators. It is suitable for comprehensive evaluation scenarios where indicators are relatively independent. The Game Theory (GT) is a combined weighting method. Its principle is to integrate subjective and objective weights within a game-theoretic framework to obtain an optimal compromise solution (Li et al., 2023).

By calculating the geometric mean of subjective and objective weights, the combined weights consider both the subjective judgment based on expert experience and the objective information driven by data, achieving a game-theoretic equilibrium between subjective preferences and objective facts. It effectively reduces the bias of single weighting and enhances the robustness and interpretability of the weights. The Rank-Sum Ratio (RSR) is a comprehensive evaluation method that combines simplicity and practicality. It is suitable for comprehensive evaluation scenarios with multiple indicators and samples, and it fits the integration and analysis of multi-dimensional crop data in agricultural production (Hao et al., 2019).

In this study, AHP was used for subjective weighting, the CV method for objective weighting, and GT was introduced as a combined weighting method. This combined weighting method, based on game-theoretic reasoning, aims to harmonize the differences in weight between subjective experience and objective data. The optimal combination coefficient is obtained via least-squares optimization, respecting expert experience while remaining faithful to the intrinsic differences in the data, thereby enhancing the robustness of the evaluation system. Subsequently, an AHP-CV-RSR evaluation model was established based on the combined weights. The treatments and their indicators were ranked, and weighted RSRs were calculated. The comprehensive evaluation of the plans was achieved through RSR value ranking and classification. The higher the RSR value, the better the plan. This model effectively integrates subjective judgment and objective information and is suitable for agricultural management or technology plan selection problems with limited resources and diverse criteria.

1. Determination of evaluation indicators and data:

In this study, an indicator set I = {i_1_, i_2_,…, i_n_} was first established to evaluate irrigation treatment schemes. These indicators encompass growth metrics, physiological indicators, resource efficiency, and output value. For each irrigation treatment scheme set J = {j_1_, j_2_,…, j_m_}, data D_ij_ were collected across these indicators.

**Figure 2:**
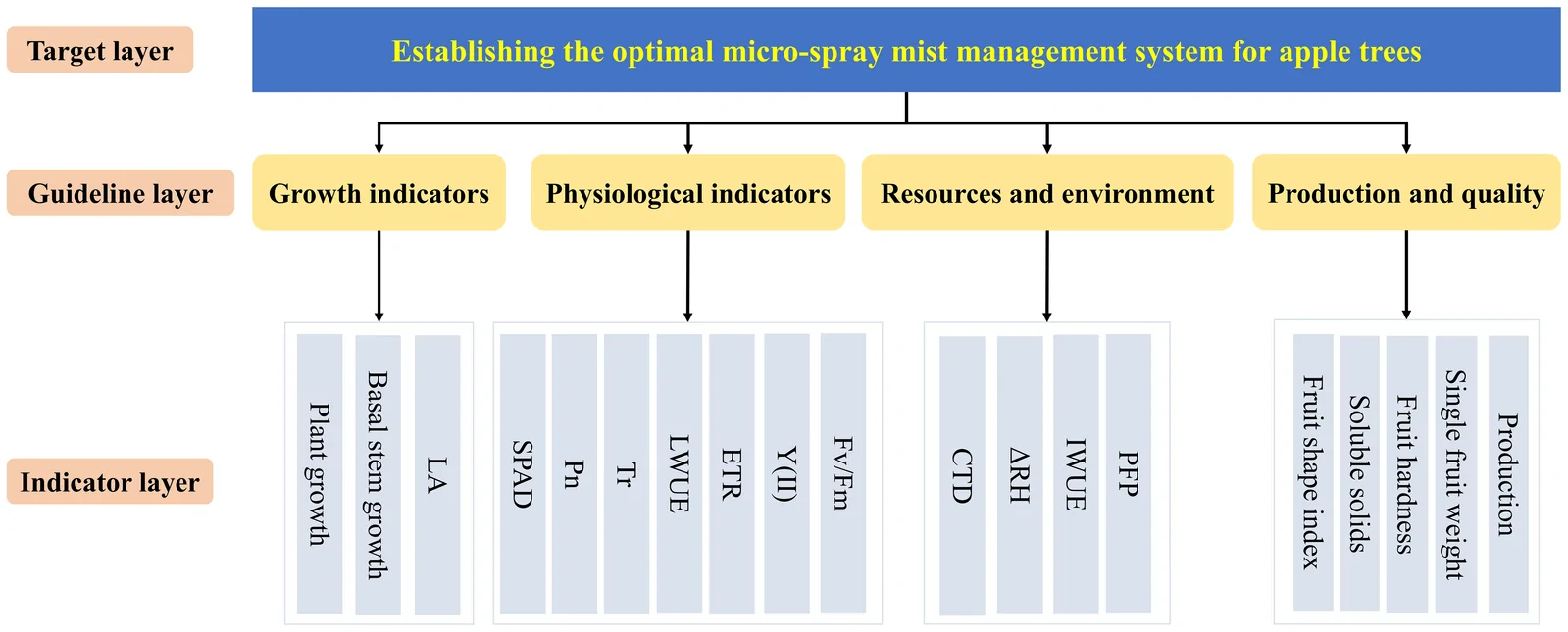
Establishing a hierarchical analytic evaluation system for optimal micro-spray misting management in apple orchards.

2. Calculation of subjective weights (AHP):

Based on the objectives selected for determining the optimal micro-spray misting management system in the experiment, an evaluation system was constructed using the AHP, as shown in **Figure 2**.

First, to eliminate the impact of different units, the original data were normalized using min-max normalization.

Based on the AHP evaluation system, the judgment matrix was constructed using Saaty’s 1–9 scale method (**Table 2**).

**Table 2:** Saaty 1–9 ratio scale method scale values.

Relative importance	Definition
1	Equally important
3	Slightly more important
5	Significantly more important
7	Clearly more important
9	Absolutely more important
2, 4, 6, 8	An intermediate value between two adjacent judgments
1/3	Slightly less important
1/5	Significantly less important
1/7	Clearly less important
1/9	Absolutely less important
1/2, 1/4, 1/6, 1/8	An intermediate value between two adjacent judgments

The judgment matrices were developed based on independent consultations with three domain experts specializing in orchard irrigation management and fruit tree physiology. Each expert independently assigned importance scores for all pairwise indicator comparisons following the 1–9 scale. The final judgment matrix used for weight calculation was derived by averaging the scoring results across all three experts.

(5) $$A={\left(a_{ij}\right)}_{m\times n}$$

In the above formula, *A* is a positive reciprocal matrix, and *a_ij_* represents the importance of the indicator *a_i_* relative to *a_j_*, which is the quantified value of the judgment matrix.

Calculation of weights:

a. The geometric mean of each row of the judgment matrix.

(6) $$\bar{u_{ı}}=\sqrt[{n}]{\prod_{j=1}^{n}a_{ij}},\;i=1,2,3,\ldots ,n$$

b. Normalization process.

(7) $$u_{i}=\frac{\bar{u_{i}}}{\sum_{i=1}^{n}\bar{u_{i}}},\;i=1,2,3,\ldots ,n$$

c. Calculation of the maximum eigenvalue of the matrix.

(8) $$\lambda_{max}=\sum_{i=1}^{n}\frac{{\left(Au\right)}_{i}}{nu_{i}}$$

d. Consistency test of the judgment matrix.

(9) $$\begin{array}{c} CR=\frac{CI}{RI}\\ CI=\frac{\lambda_{max}-n}{n-1} \end{array}$$

In the above formula, CR is the random consistency ratio; CI is the consistency index; RI is the average random consistency index. If CR< 0.1, the judgment matrix has satisfactory consistency.

3. Calculation of subjective weights using the CV method:

Standardize the data:

(10) $$D_{ij}^{'}=\frac{D_{ij}-\mu_{j}}{\sigma_{j}}$$

Where *μ_j_* and σ*_j_* are the mean and standard deviation of indicator j, respectively.

Calculate the CV:

(11) $$CV_{j}=\frac{\sigma_{j}}{\mu_{j}}$$

Determine objective weights based on the CV:

(12) $$w_{i}^{o}=\frac{\frac{1}{CV_{j}}}{\sum_{k-1}^{n}\frac{1}{CV_{k}}}$$

4. Game theory calculation of weighted combinations:

Treating the subjective and objective weights as game players, first define α = {α_1_, α_2_} as the linear combination coefficients. The linear combination of the two weight vectors is then:

(13) $$W=\;\alpha_{1}w_{1}^{T}+\;\alpha_{2}w_{2}^{T}$$

Based on the game-theoretic aggregation model, the two linear combination coefficients are optimized to minimize deviation and obtain the most satisfactory weights in W. The objective function is established as follows:

(14) $$min{\left\|\sum_{p=1}^{n}\alpha_{p}w_{p}^{T}-w_{p}\right\|}_{2}$$

In the equation, $\sum_{\text{p}=1}^{\text{n}}\alpha_{\text{p}}{\text{w}}_{\text{p}}^{\text{T}}$ denotes the vector set of summation-weighted components.

Applying matrix differential properties, the above expression is equivalently transformed into a linear system of equations for the first-order derivative conditions of the optimization problem:

(15) $$\left[\begin{array}{cc} w_{1}w_{1}^{T} & w_{1}w_{2}^{T}\\ w_{2}w_{1}^{T} & w_{2}w_{2}^{T} \end{array}\right]\left[\begin{array}{c} \alpha_{1}\\ \alpha_{2} \end{array}\right]=\left[\begin{array}{c} w_{1}w_{1}^{T}\\ w_{2}w_{2}^{T} \end{array}\right]$$

Solve for the optimized combination coefficients α_1_ and α_2_, then perform normalization:

(16) $$\{\;\begin{array}{l} \alpha_{1}^{*}=\frac{\alpha_{1}}{\left(\alpha_{1}+\alpha_{2}\right)}\\ \alpha_{2}^{*}=\frac{\alpha_{2}}{\left(\alpha_{1}+\alpha_{2}\right)} \end{array}$$

The final composite weighting of the indicators is obtained using Equation 17.

(17) $$W=\alpha_{1}^{*}w_{1}^{T}{+}_{2}^{*}w_{2}^{T}$$

5. RSR comprehensive evaluation method:

Based on the data for each indicator, rank each micro-sprinkler misting treatment scheme. Select the “non-integer ranking method” to distinguish differences between schemes more precisely. Apply combined weights to perform a weighted summation of rank sums, calculating the weighted RSR value:

For benefit- and cost-based indicators, the rank values were calculated using Equations 18, 19, respectively:

(18) $$R_{ij}=1+\left(n-1\right)\frac{X_{ij}-min\left(X_{1j},X_{2j},\ldots ,X_{nj}\right)}{max\left(X_{1j},X_{2j},\ldots ,X_{nj}\right)-min\left(X_{1j},X_{2j},\ldots ,X_{nj}\right)}$$

(19) $$R_{ij}=1+\left(n-1\right)\frac{max\left(X_{1j},X_{2j},\ldots ,X_{nj}\right)-X_{ij}}{max\left(X_{1j},X_{2j},\ldots ,X_{nj}\right)-min\left(X_{1j},X_{2j},\ldots ,X_{nj}\right)}$$

Where *n* represents the number of treatments and *m* denotes the number of evaluation indicators.

Calculate the RSR value:

When weights are equal, the RSR value was calculated using Equation 20:

(20) $$RSR_{i}=\frac{1}{n\times m}\sum_{j=1}^{m}R_{ij}$$

When weights differ, the weighted RSR value was calculated using Equation 21:

(21) $$WRSR_{i}=\frac{1}{n}\sum_{j=1}^{m}W_{j}R_{ij}$$

R_ij_ denotes the rank of the jth indicator for the ith object, while W_j_ represents the weight of the jth indicator, with the sum of weights equaling 1. A higher value of RSR_i_ indicates a superior evaluation object.

6. Classification and evaluation

Based on the RSR values, the micro-spray misting treatment plans are divided into different categories: high (excellent), medium, and low (poor). The best micro-spray misting treatment is determined according to the RSR values and classification results. The treatment plan with the highest RSR value is usually considered the best irrigation treatment plan.

Through the above steps, different micro-spray misting plans can be systematically evaluated, and the best micro-spray misting treatment can be determined to improve apple quality and yield and to improve the field microclimate. This method combines expert knowledge, data-driven analysis, and comprehensive evaluation to provide scientific and rational decision-making support.

### 2.5 Data processing

Data were organized using Microsoft Excel 2019. Statistical analyses, including variance analysis and multiple comparisons, were performed using SPSS software (IBM, USA). A comprehensive evaluation was conducted using SPSSPRO (online platform: https://www.spsspro.com) and Python. Graphical visualizations were created with Origin 2025 and ArcGIS Pro.

## 3 Results

### 3.1 Effects of different micro-spray misting treatments on apple growth

Based on a systematic evaluation of different micro-spray treatments on plant growth and basal stem growth (**Table 3**), the results revealed that in 2024, only misting height (H) exerted a highly significant effect on basal stem growth throughout the growth period (P< 0.01), while misting duration (T) and their interaction (H × T) had no significant effects (P > 0.05). In 2025, none of the factors significantly influenced basal stem growth (P > 0.05). Regarding plant growth, micro-spray height had a significant effect on plant growth throughout the entire growth period in 2024 (P< 0.05), while misting duration and the H × T interaction exerted highly significant effects (P< 0.01). In 2025, micro-spray height, misting duration, and their interaction all reached highly significant levels for plant growth (P< 0.01).

**Table 3:** Results of two-way analysis of variance (ANOVA) for the effects of misting height (H), misting duration (T), and their interaction (H×T) on plant growth and basal stem growth during 2024–2025.

Factor	*F* value (Plant growth)		*F* value (Basal stem growth)	
	2024	2025	2024	2025
H	21.7*	1892.09**	33.01**	4.70^ns^
T	187.47**	58.71**	1.24 ^ns^	0.28^ns^
H×T	25.65**	59.56**	2.04 ^ns^	1.05^ns^

*P<0.05 (significant difference), **P<0.01 (highly significant difference), ns (not significant). This notation is consistent throughout the paper.

**Figure 3:**
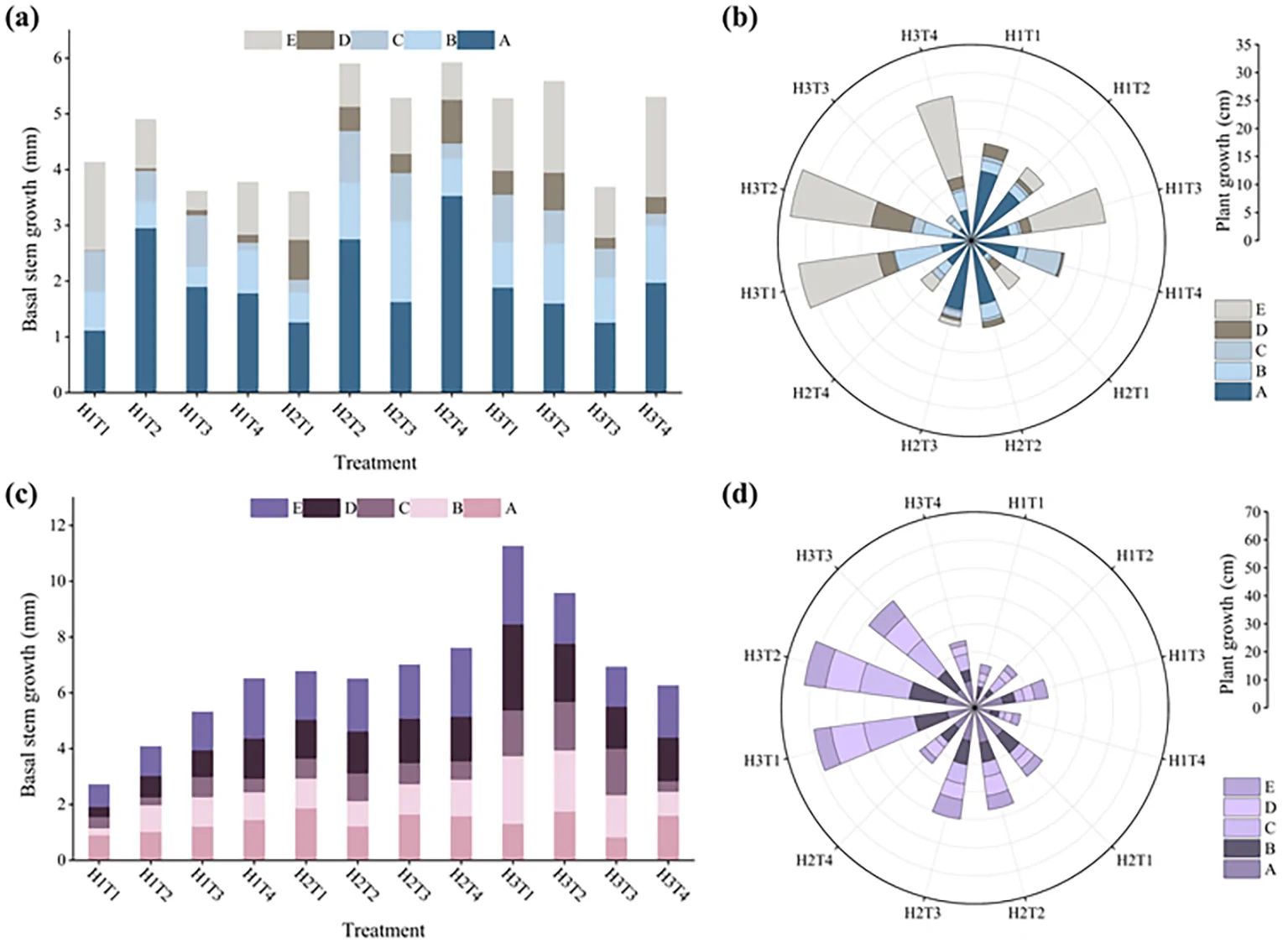
Effects of different treatments on apple tree growth in 2024 and 2025. **(a, b)** Data for 2024; **(c, d)** data for 2025. **(a, c)** Stacked bar charts showing basal stem growth contributions at each phenological stage under treatments H1T1–H3T4. **(b, d)** are stacked polar area charts illustrating the plant growth contribution at each phenological stage. A, B, C, D, and E represent the bud break and flowering stage, the shoot growth stage, the fruit setting stage, the fruit enlargement stage, and the fruit maturity stage, respectively. Each colored segment indicates the contribution of a given phenological stage to the total growth under a specific treatment.

As shown in **Figure 3**, in 2024, the first year of micro-spray misting treatment, the overall basal stem growth was H2 > H3 > H1. The basal stem growth of the middle canopy treatment (H2) was 30.20% and 5.85% higher than that of the upper canopy (H1) and lower canopy (H3) treatments, respectively. The maximum basal stem growth was observed in the H2T2 treatment, at 5.90 mm. In 2025, the second year of micro-spray misting treatment, the overall basal stem growth was H3 > H2 > H1. The basal stem growth of the lower canopy treatment (H3) was 78.88% and 29.55% higher than that of the upper canopy (H1) and middle canopy (H2) treatments, respectively. The maximum basal stem growth was observed in the H3T1 treatment, at 10.49 mm. In 2024, the first year of micro-spray misting treatment, the overall plant growth was H3 > H1 > H2. The plant growth of the lower canopy treatment (H3) was 27.57% and 79.07% higher than that of the upper canopy (H1) and middle canopy (H2) treatments, respectively. The maximum plant growth was observed in the H3T2 treatment, at 33.00 cm. In 2025, the second year of micro-spray misting treatment, the overall plant growth was H3 > H2 > H1. The plant growth of the lower canopy treatment (H3) was 146.22% and 45.60% higher than that of the upper canopy (H1) and middle canopy (H2) treatments, respectively. The maximum plant growth under the interaction of micro-spray height and misting duration was observed in the H3T2 treatment, at 62.15 cm. In addition, the plant growth and basal stem growth in all treatments in 2025 were generally higher than those in 2024, which may be related to the increase in tree age.

**Figure 4:**
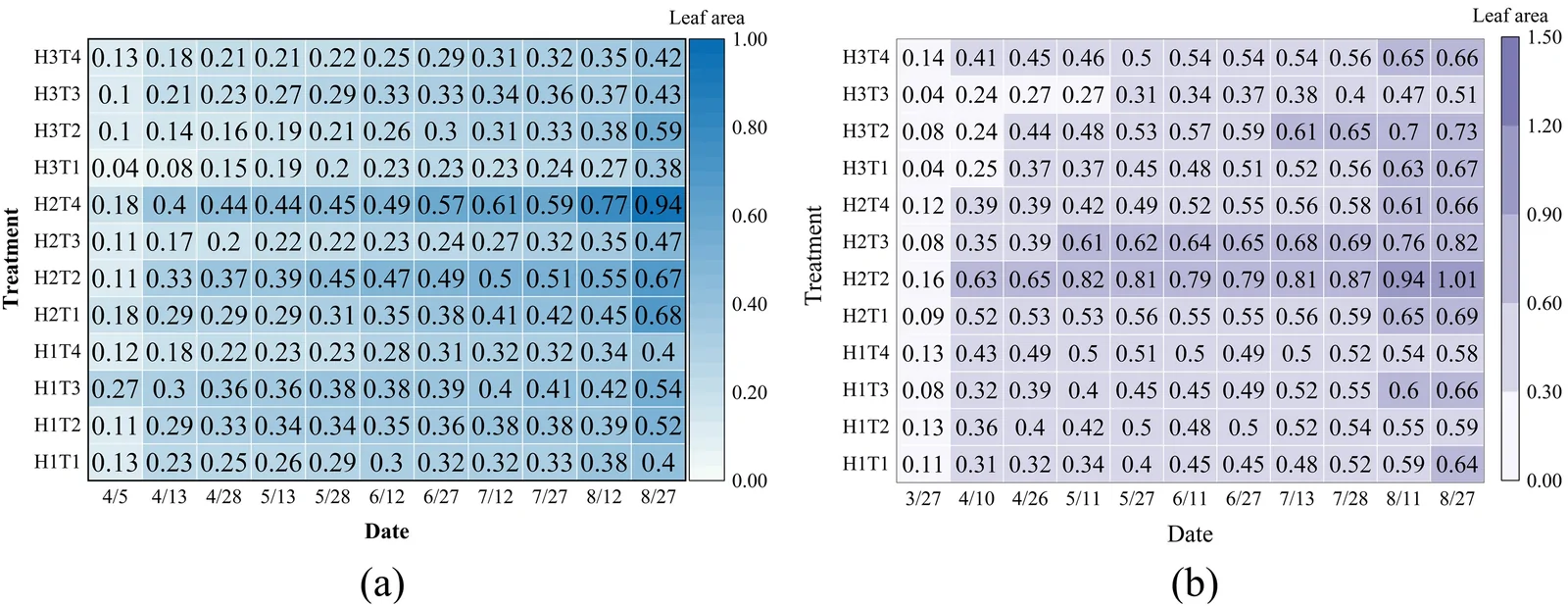
The effect of different treatments on apple leaf area in 2024 **(a)** and 2025 **(b)**.

Based on the leaf area data measured in 2024 and 2025, the impact of different micro-spray misting treatments on apple leaf area was analyzed. It was found that only misting height (H) had a significant effect on leaf area in 2025 (P< 0.05), while misting duration (T) and its interaction with micro-spray height did not significantly affect leaf area in either 2024 or 2025 (**Table 4**). As shown in **Figure 4**, in the first year of micro-spray misting treatment in 2024, the overall leaf area was H2 > H1 ≈ H3. The middle canopy treatment (H2) had a leaf area 48.39% and 51.65% higher than the upper canopy (H1) and lower canopy (H3) treatments, respectively. The maximum leaf area under the interaction of micro-spray height and misting duration was observed in the H2T4 treatment, at 0.94 m². In the second year of micro-spray misting treatment in 2025, the overall leaf area was H2 > H3 ≈ H1. The middle canopy treatment (H2) had a leaf area 28.20% and 23.27% higher than the upper canopy (H1) and lower canopy (H3) treatments, respectively. The maximum leaf area under the interaction of micro-spray height and misting duration was observed in the H2T4 treatment, at 1.01 m². In addition, the leaf area of all treatments in 2025 was generally higher than that in 2024, which may be related to tree age and the sufficient rainfall in 2025. These results indicate that misting in the middle canopy can promote leaf growth and development, and this effect shows relative stability across different years.

**Table 4:** Results of two-way ANOVA for leaf area from 2024 to 2025.

Factors	*F* value (Leaf area)	
	2024	2025
H	3.15^ns^	5.68*
T	0.47^ns^	1.66^ns^
H×T	1.85^ns^	1.23^ns^

### 3.2 Effects of micro-spray misting on the physiological characteristics of apple trees

#### 3.2.1 Chlorophyll SPAD value

**Figure 5:**
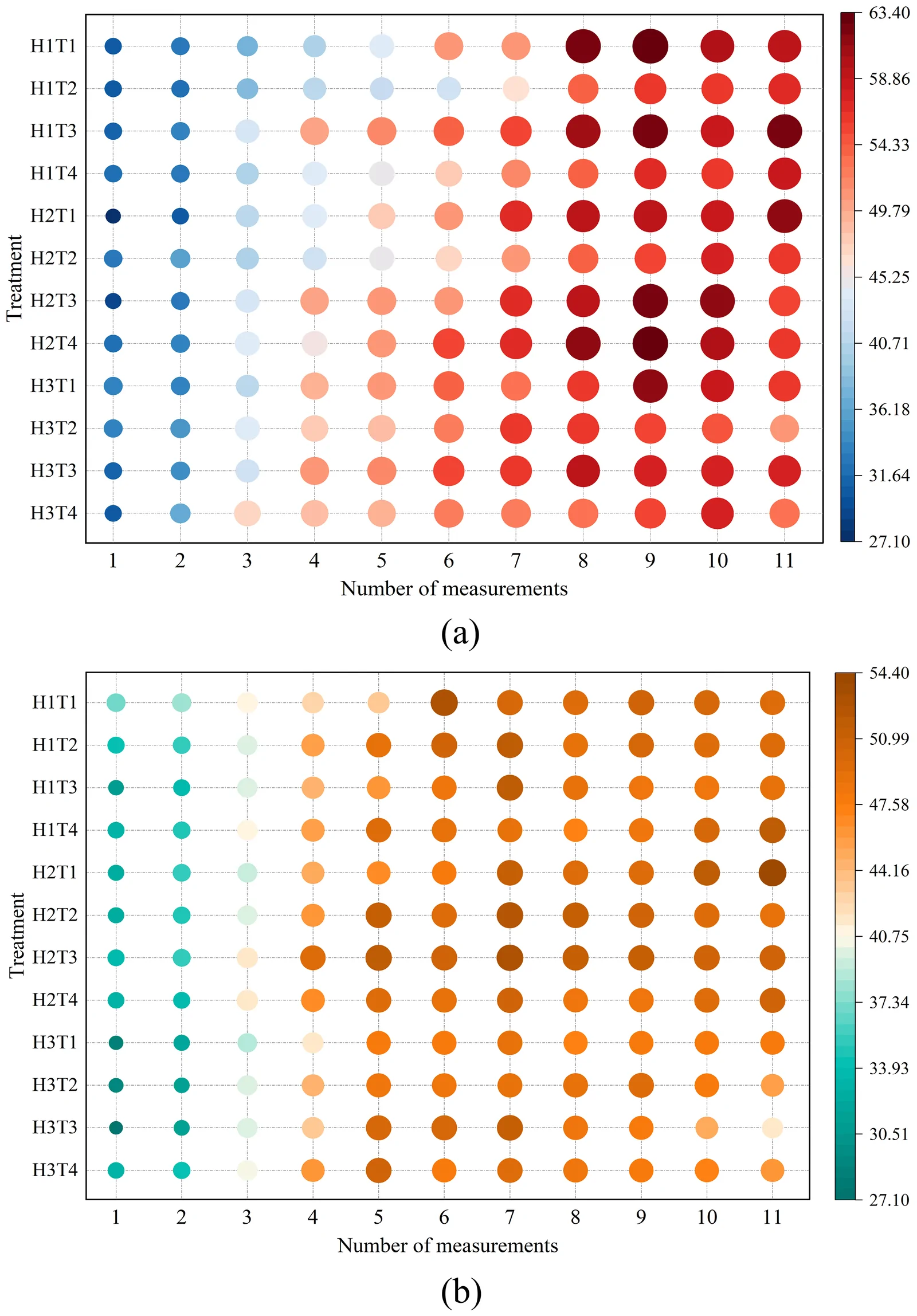
The impact of different treatments on Relative chlorophyll content (SPAD) in 2024 **(a)** and 2025 **(b)**.

Chlorophyll is a key pigment for photosynthesis in plants, and its content directly affects the efficiency of light energy capture and carbon assimilation. Over the two consecutive years of experiments from 2024 to 2025, as the growing season progressed, the SPAD value exhibited a trend of “initial increase, followed by a decrease, and then stabilization.” The rapid growth period of SPAD in young apple trees occurred in the early growing season (**Figure 5**). It is worth noting that there were no significant differences among the treatments, and the overall SPAD values in 2025 were slightly lower than those in 2024. In 2024, the overall SPAD values of the first-year micro-spray misting treatment were H2 ≈ H3 > H1. The SPAD value of the H1 treatment in the upper canopy was 2.55% and 3.00% lower than those of the H2 and H3 treatments in the middle and lower canopy, respectively. The maximum SPAD value in 2024 was recorded in the H1T1 treatment, at 63.33. In 2025, the overall SPAD values of the second-year micro-spray misting treatment were H2 ≈ H1 > H3. The SPAD value of the H3 treatment in the lower canopy was 3.58% and 4.99% lower than those of the H1 and H2 treatments in the upper and middle canopies, respectively. The maximum SPAD value in 2025 was recorded in the H2T1 treatment, at 54.35, which was 8.42% higher than the 49.30 of the H1T1 treatment. The middle canopy treatment exhibited higher SPAD values in both years of the experiment, indicating that micro-spray misting in the middle canopy is more conducive to chlorophyll synthesis and that this promoting effect becomes even more pronounced after a year of the experimental treatment.

#### 3.2.2 Photosynthetic indices

**Figure 6:**
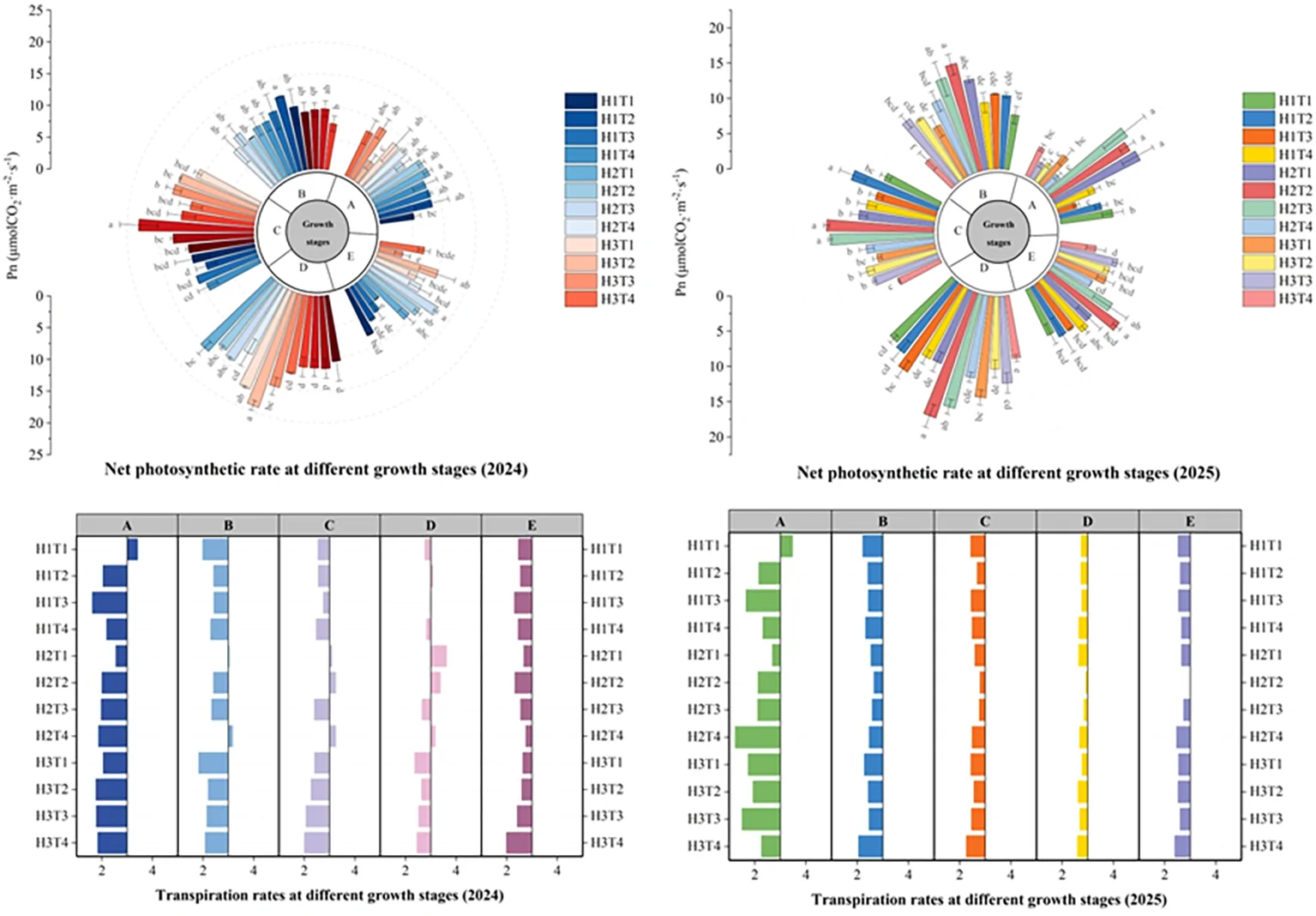
Effects of micro-spray misting on apple photosynthetic characteristics. A, B, C, D, and E represent the bud break and flowering stage, the shoot growth stage, the fruit setting stage, the fruit enlargement stage, and the fruit maturity stage, respectively.

To reveal the response patterns of apple photosynthetic parameters to micro-spray misting, analyses were conducted on key photosynthetic indices, including net photosynthetic rate (Pn), transpiration rate (Tr), and LWUE. These photosynthetic indices of apples showed distinct spatiotemporal differences in response to micro-spray misting treatments. As shown in **Figure 6**, in the first year of micro-spray misting treatment in 2024, both Pn and Tr generally exhibited the pattern of H2 > H1 > H3. The average Pn of the middle canopy treatment (H2) was 21.03% and 33.36% higher than that of the upper canopy (H1) and lower canopy (H3) treatments, respectively. The maximum Pn value in 2024 was observed in the H2T2 treatment, at 19.06 μmol m^−^² s^−1^. The average Tr of the middle canopy treatment (H2) was 8.70% and 23.78% higher than that of the upper canopy (H1) and lower canopy (H3) treatments, respectively. The maximum Tr value in 2024 was observed in the H2T1 treatment, at 3.63 mmol m^−^² s^−1^. In the second year of micro-spray misting treatment in 2025, both Pn and Tr generally exhibited the pattern of H2>H1>H3. The average Pn of the middle canopy treatment (H2) was 31.78% and 51.27% higher than that of the upper canopy (H1) and lower canopy (H3) treatments, respectively. The maximum Pn value in 2025 was observed in the H2T2 treatment, at 18.74 μmol m^−^² s^−1^. The average Tr of the middle canopy treatment (H2) was 1.45% and 8.22% higher than that of the upper canopy (H1) and lower canopy (H3) treatments, respectively. The maximum Tr value in 2025 was observed in the H1T1 treatment, at 3.27 mmol m^−^² s^−1^. In addition, Apple Pn in 2025 showed a slight overall decline compared to 2024. This change is consistent with the interannual dynamics of the aforementioned SPAD values, indicating an intrinsic consistency between chlorophyll content fluctuations and changes in photosynthetic capacity. It further confirms the important regulatory role of leaf pigment levels on the efficiency of light energy conversion. The effects of different treatments on LWUE in 2024–2025 are shown in **Table 5**. In the first year of micro-spray misting treatment in 2024, LWUE generally exhibited the pattern of H3 > H2 > H1. The average LWUE of the lower canopy treatment (H3) was 38.91% and 8.72% higher than that of the upper canopy (H1) and middle canopy (H2) treatments, respectively. The maximum LWUE value in 2024 was observed in the H2T3 treatment, at 5.73 μmol mmol^−1^. In the second year of micro-spray misting treatment in 2025, LWUE generally exhibited the pattern of H2 > H1 > H3. The average LWUE of the middle canopy treatment (H2) was 30.58% and 43.02% higher than that of the upper canopy (H1) and lower canopy (H3) treatments, respectively. The maximum LWUE value in 2025 was observed in the H2T2 treatment, at 6.40 μmol mmol^−1^.

**Table 5:** Leaf instantaneous water use efficiency (LWUE) of apple trees under different micro-spray mist treatments across five growth stages in 2024 and 2025.

Height	Duration	A	B	C	D	E
LWUE in 2024 (μmol mmol^-1^)						
H1	T1	1.59 ± 0.37c	3.72 ± 0.83a	4.34 ± 0.35abcd	5.62 ± 0.41abc	3.29 ± 0.07bc
	T2	4.34 ± 1.88ab	3.96 ± 0.02a	5.26 ± 0.92ab	4.36 ± 0.19bcd	2.52 ± 0.56cd
	T3	5.40 ± 0.10a	3.90 ± 0.80a	4.95 ± 0.11abc	4.74 ± 0.17abcd	2.40 ± 0.49cd
	T4	3.94 ± 0.02abc	4.01 ± 0.99a	4.21 ± 0.17abcd	4.21 ± 0.94d	1.65 ± 0.15d
H2	T1	3.97 ± 1.01abc	3.37 ± 1.04a	3.72 ± 0.31bcd	4.66 ± 0.27abcd	3.48 ± 0.20abc
	T2	4.87 ± 0.49a	5.09 ± 0.20a	5.62 ± 0.19a	5.65 ± 0.24ab	4.41 ± 0.24ab
	T3	3.71 ± 0.86abc	4.49 ± 1.70a	5.34 ± 0.42a	5.73 ± 0.13a	4.94 ± 0.80a
	T4	4.71 ± 0.32ab	2.91 ± 0.37a	3.22 ± 0.01d	3.96 ± 0.29d	3.11 ± 0.94bcd
H3	T1	4.23 ± 1.97ab	5.06 ± 0.84a	4.29 ± 1.54abcd	4.79 ± 0.25abcd	2.68 ± 0.34cd
	T2	2.25 ± 0.70bc	3.73 ± 0.21a	3.65 ± 0.26cd	4.33 ± 0.65bcd	3.86 ± 1.13abc
	T3	5.24 ± 0.67a	4.67 ± 1.43a	5.00 ± 0.32abc	4.59 ± 0.91abcd	1.61 ± 0.54d
	T4	4.28 ± 1.29ab	3.96 ± 1.26a	4.64 ± 0.98abcd	4.27 ± 1.06cd	3.68 ± 1.21abc
*F* value						
H		0.68^ns^	1.46 ^ns^	0.59 ^ns^	1.49 ^ns^	104.05**
T		4.27^ns^	0.30 ^ns^	4.80 ^ns^	4.47 ns	2.01 ns
H×T		2.17^ns^	1.85 ^ns^	2.40 ^ns^	1.71 ^ns^	2.70 ^ns^
LWUE in 2025 (μmol mmol^-1^)						
H1	T1	2.28 ± 0.26b	3.53 ± 0.35e	3.61 ± 0.31bc	4.43 ± 0.13cd	2.87 ± 0.57bcd
	T2	3.16 ± 0.85b	4.34 ± 0.13cde	4.88 ± 0.56a	4.66 ± 0.59cd	3.12 ± 0.88abcd
	T3	1.60 ± 0.39b	4.38 ± 0.09cde	3.69 ± 0.36b	5.27 ± 0.44bc	3.21 ± 0.03abcd
	T4	2.89 ± 0.83b	4.08 ± 0.38de	4.02 ± 0.49b	4.29 ± 0.27de	3.57 ± 0.17abc
H2	T1	5.51 ± 0.31a	5.16 ± 0.11abc	4.18 ± 0.26b	4.26 ± 0.40de	3.28 ± 0.64abc
	T2	5.96 ± 0.27a	5.92 ± 0.41a	5.48 ± 0.12a	6.40 ± 0.50a	4.16 ± 0.03a
	T3	6.10 ± 2.43a	5.48 ± 0.75ab	5.36 ± 0.08a	5.84 ± 0.23ab	3.67 ± 0.63ab
	T4	2.09 ± 0.41b	4.68 ± 0.53bcd	3.93 ± 0.32b	4.43 ± 0.22cd	2.46 ± 0.01cd
H3	T1	2.76 ± 0.30b	3.67 ± 0.48de	3.48 ± 0.22bc	5.23 ± 0.33bc	3.03 ± 0.45bcd
	T2	1.80 ± 0.56b	4.33 ± 0.02cde	4.05 ± 0.20b	3.96 ± 0.44de	3.03 ± 0.26bcd
	T3	1.69 ± 0.57b	4.64 ± 0.32bcd	3.99 ± 0.10b	4.59 ± 0.44cd	3.25 ± 0.25abc
	T4	2.25 ± 0.59b	2.55 ± 0.69f	2.93 ± 0.03c	3.47 ± 0.18e	2.13 ± 0.51d
*F* value						
H		24.28*	47.81*	63.01*	17.44^ns^	3.97^ns^
T		0.94^ns^	7.33^ns^	37.31^ns^	7.59^ns^	2.28^ns^
H×T		13.41**	2.04^ns^	4.21^ns^	12.45**	3.10^ns^

Growth stages are denoted as A = bud break and flowering stage, B = shoot growth stage, C = fruit setting stage, D = fruit enlargement stage, and E = fruit maturity stage.

#### 3.2.3 Chlorophyll fluorescence parameters

Based on the core parameters of chlorophyll fluorescence Y(II), ETR, and Fv/Fm during the key growth stages of apples from 2024 to 2025, an in-depth analysis was conducted on the regulatory effects of micro-spray misting on the function of the photosynthetic system. Analysis of variance showed (**Table 6**) that the effects of micro-spray misting on chlorophyll fluorescence parameters were not significant in most periods. In 2024, only during the bud break and flowering stages, H and T had significant effects on Fv/Fm (P< 0.05), and the interaction between H and T (H × T) had a significant effect on Y(II) (P< 0.05). In 2025, the regulatory effects in different growth stages were further enhanced. During the bud break and flowering stage, H had a significant effect on Y(II) (P< 0.05); during the fruit setting stage, T had a significant effect on ETR, and H×T had a significant effect on Y(II) (P< 0.05); during the fruit enlargement stage, H had a significant effect on ETR, and H×T had significant effects on Y(II) and Fv/Fm (P< 0.05).

**Table 6:** ANOVA for chlorophyll fluorescence parameters during key growth stages of apple trees in 2024-2025.

Factor	*F* value [Y(II)]						*F* value (ETR)						*F* value (Fv/Fm)					
	2024			2025			2024			2025			2024			2025		
	A	C	D	A	C	D	A	C	D	A	C	D	A	C	D	A	C	D
H	ns	ns	ns	*	ns	ns	ns	ns	ns	ns	ns	*	*	ns	ns	ns	ns	ns
T	ns	ns	ns	ns	ns	ns	ns	ns	ns	ns	*	ns	*	ns	ns	ns	ns	ns
H×T	*	ns	ns	ns	*	*	ns	ns	ns	ns	ns	ns	ns	ns	ns	ns	ns	*

Growth stages are denoted as A = bud break and flowering stage, C = fruit setting stage, D = fruit enlargement stage.

**Figure 7:**
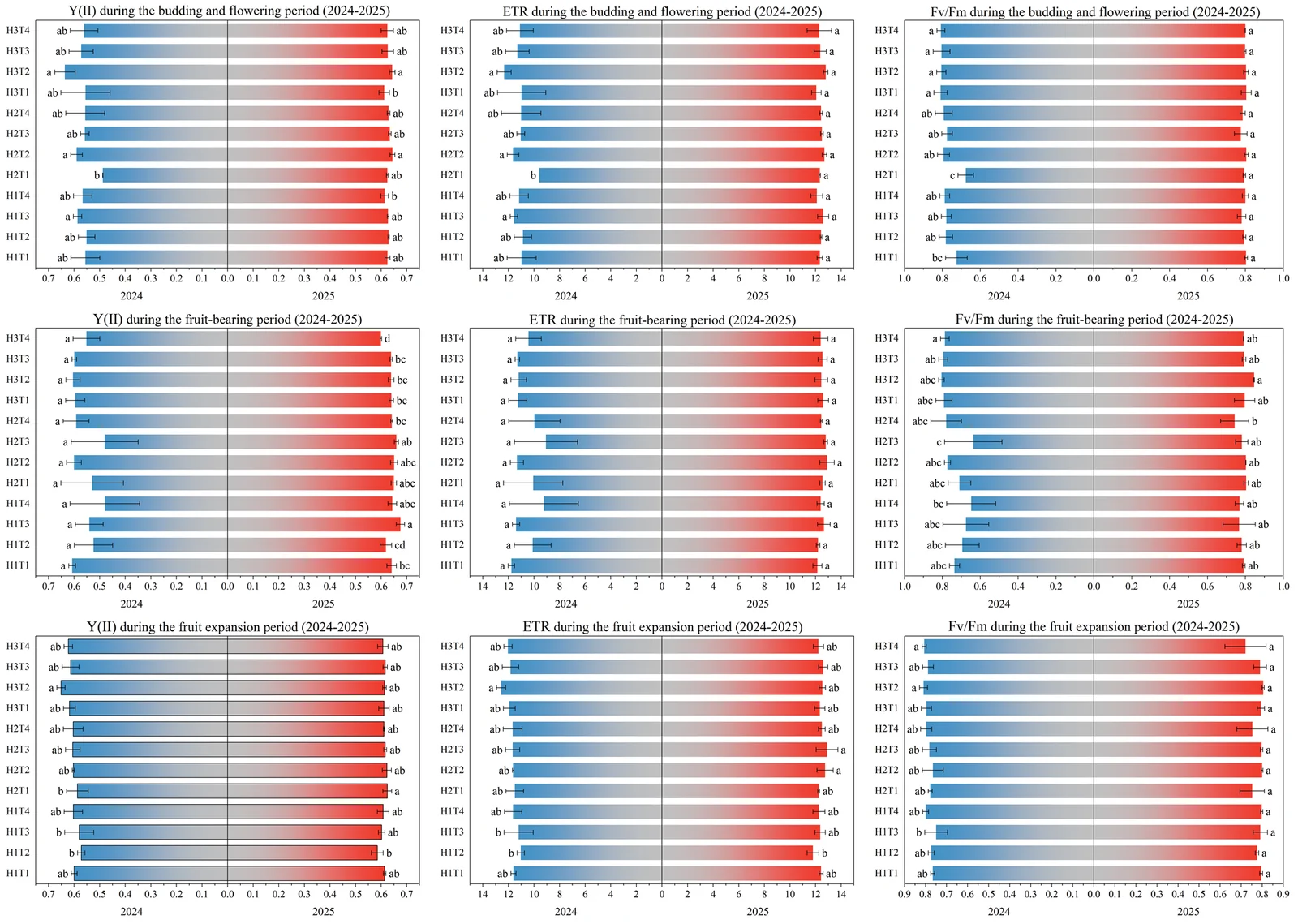
The impact of different treatments on chlorophyll fluorescence parameters during key growth stages of apples in 2024–2025.

As shown in **Figure 7**, during each key growth stage, in the first year of micro-spray misting treatment in 2024, Y(II) generally followed the pattern H3 > H2 > H1. The average Y(II) of the lower canopy treatment (H3) was 6.12% and 5.17% higher than that of the upper canopy (H1) and middle canopy (H2) treatments, respectively. The maximum Y(II) value in 2024 was observed in the H3T2 treatment, at 0.65. ETR generally exhibited the pattern of H1 ≈ H3 > H2. The average ETR of the upper canopy treatment (H1) was 12.83% and 0.32% higher than that of the middle canopy (H2) and lower canopy (H3) treatments, respectively. The maximum ETR value in 2024 was observed in the H3T2 treatment, at 12.57. Fv/Fm was maintained between 0.60 and 0.85, generally exhibiting the pattern of H2 > H1 > H3. The average Fv/Fm of the middle canopy treatment (H2) was 10.41% and 11.40% higher than that of the upper canopy (H1) and lower canopy (H3) treatments, respectively. The maximum Fv/Fm value in 2024 was observed in the H3T2 treatment, at 0.81. In the second year of micro-spray misting treatment in 2025, Y(II) generally exhibited the pattern of H2 > H1 > H3. The average Y(II) of the middle canopy treatment (H2) was 1.83% and 1.66% higher than that of the upper canopy (H1) and lower canopy (H3) treatments, respectively. The maximum Y(II) value in 2025 was observed in the H1T3 treatment, at 0.68. ETR generally exhibited the pattern of H2 > H3 > H1. The average ETR of the middle canopy treatment (H2) was 2.26% and 1.23% higher than that of the upper canopy (H1) and lower canopy (H3) treatments, respectively. The maximum ETR value in 2025 was observed in the H2T2 treatment, at 12.90. Fv/Fm was generally maintained between 0.72 and 0.85, exhibiting the pattern of H3>H1>H2. The average Fv/Fm of the lower canopy treatment (H3) was 1.01% and 1.07% higher than that of the upper canopy (H1) and middle canopy (H2) treatments, respectively. The maximum Fv/Fm value in 2025 was observed in the H3T2 treatment, at 0.85. From an interannual perspective, the fluorescence parameters in 2025 were higher than those in 2024, with Y(II) increasing by 8.76%, ETR by 24.72%, and Fv/Fm by 10.18%. It may be related to the long-term cumulative effects of micro-spray misting treatment.

### 3.3 Effects of different micro-spray misting treatments on fruits

**Figure 8:**
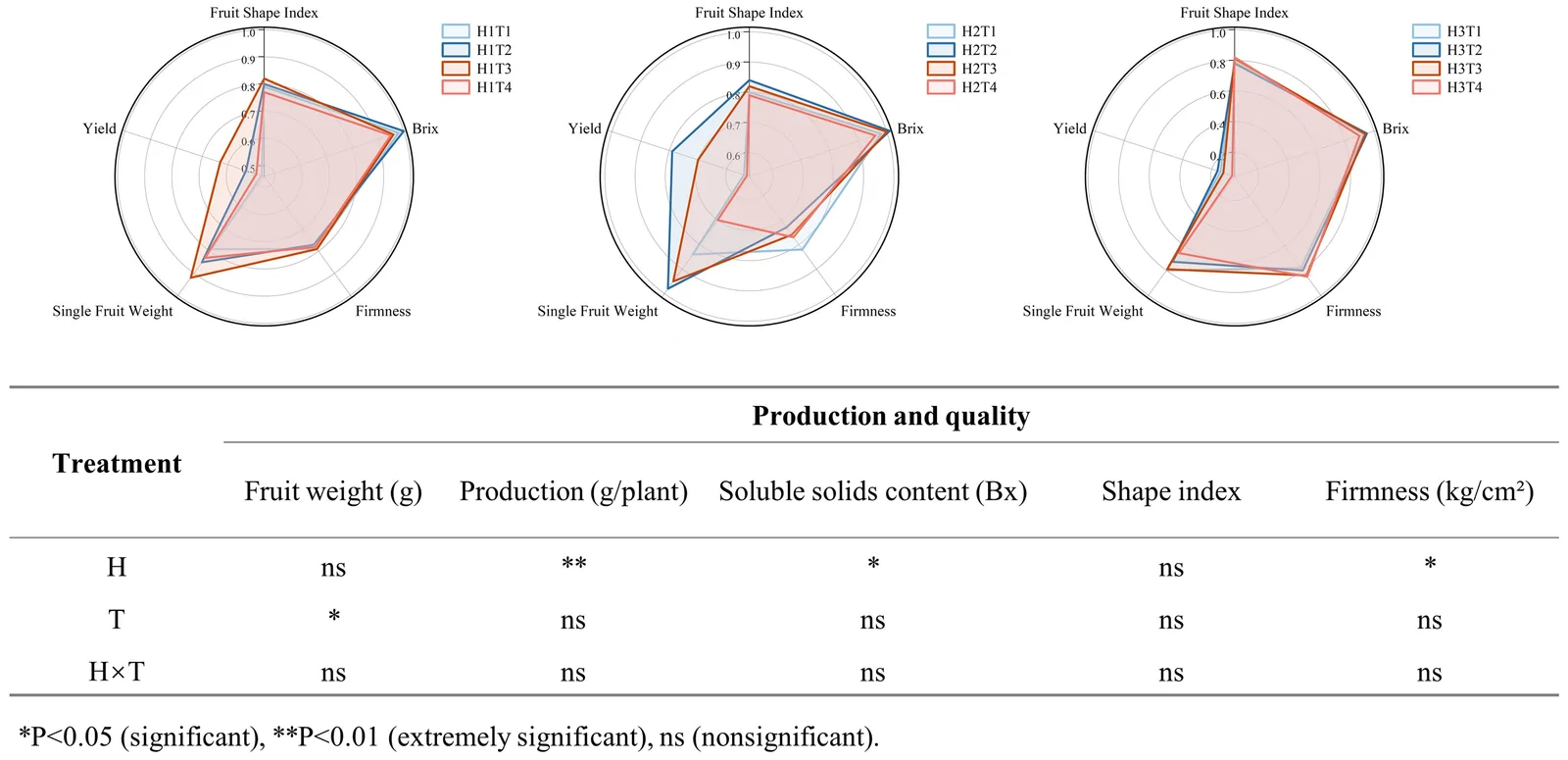
Yield and quality of fruits under different treatments.

After the fruits matured in 2025, the yield, single fruit weight, soluble solids content, fruit shape index, and hardness of each treatment were recorded for individual apple trees, with specific results shown in **Figure 8**. Micro-spray position had a highly significant effect on yield (P< 0.01) and significant effects on soluble solids content and hardness (P< 0.05), but no significant effect on fruit shape index. Misting duration had a significant effect on single fruit weight (P< 0.05). The interaction between misting duration and micro-spray position did not significantly affect any of the indices. Among them, the top three treatments for single fruit weight, in descending order, were H2T2 > H2T3> H1T3. The single fruit weight of H2T2 was 4.20% and 7.49% higher than that of H2T3 and H1T3, respectively. The top three treatments for yield, in descending order, were also H2T2 > H2T3 > H1T3. The yield of H2T2 was 5503.16 g per plant, which was 13.13% and 27.56% higher than that of H2T3 and H1T3, respectively. In addition, the H2T2 treatment had the best values for soluble solids content and hardness. There were no significant differences in fruit shape index among different treatments.

### 3.4 Water and fertilizer use efficiency, canopy temperature, and humidity

**Figure 9:**
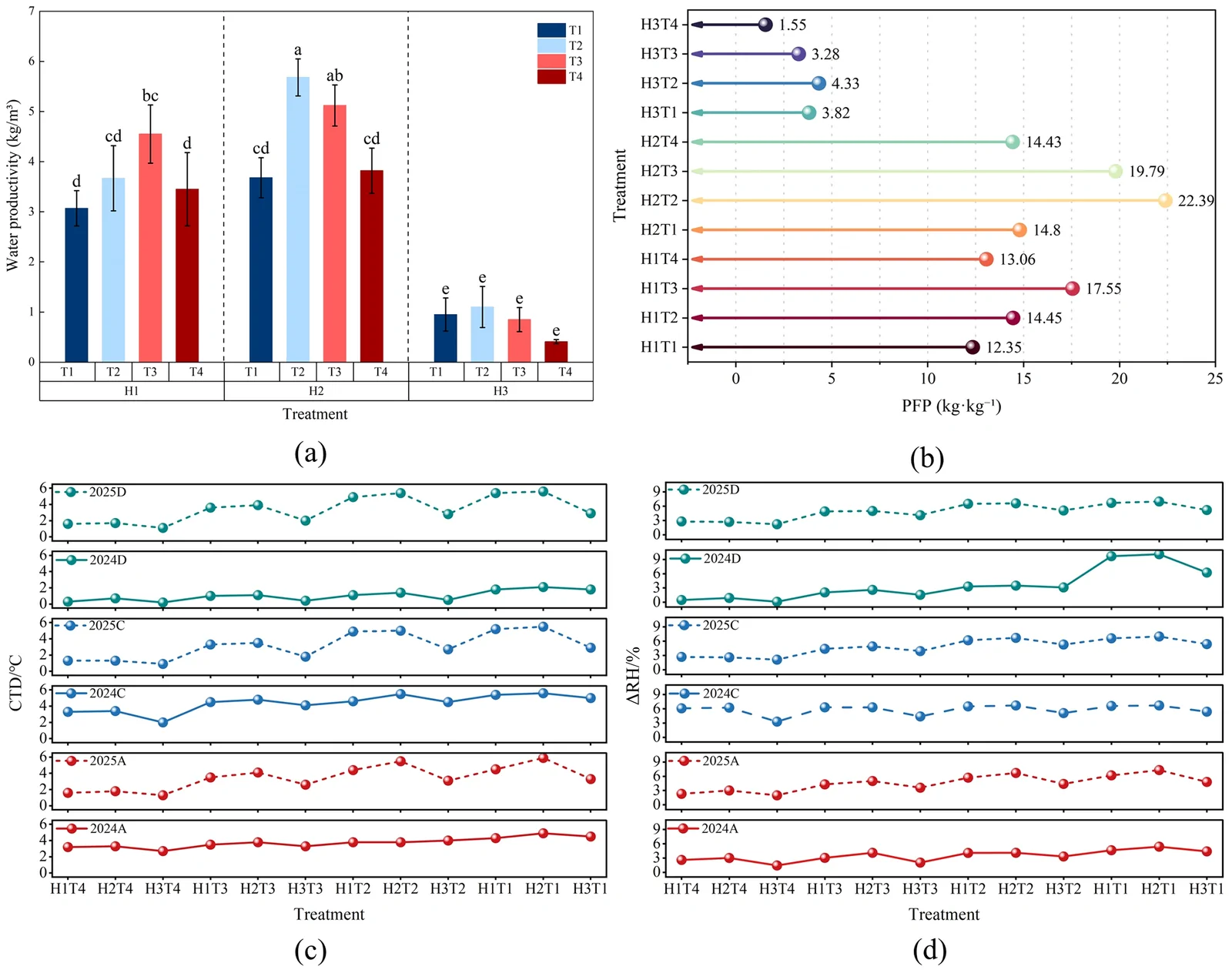
The effects of different treatments on water and fertilizer utilization efficiency and canopy temperature and humidity. Panels **(a–d)** represent water use efficiency (WUE), partial factor productivity (PFP), canopy temperature decrease (CTD), and increase in canopy relative humidity (ΔRH), respectively.

As shown in **Figure 9a**, at a certain micro-spray height, the WUE first increased and then decreased with increasing misting duration, generally following the pattern T3 > T2 > T1 > T4. The WUE of the slight misting treatment T3 was 36.62%, 0.67%, and 36.98% higher than that of the other misting treatments T1, T2, and T4, respectively. For a given misting duration, the WUE first increased and then decreased with decreasing micro-spray height, generally following the pattern H2 > H1 > H3. The WUE of the middle canopy treatment (H2) was 75.11% and 452.87% higher than that of the upper canopy (H1) and lower canopy (H3) treatments, respectively. The maximum WUE under the interaction of micro-spray height and misting duration was observed in the H2T2 treatment at 5.68 kg·m^−3^, indicating that appropriate misting in the middle canopy can yield a higher WUE. As shown in **Figure 9b**, at a given micro-spray height, the PFP of fertilizer first increased and then decreased with increasing misting duration, generally following the pattern T2 > T3 > T1 > T4. The PFP of the moderate misting treatment T2 was 32.94%, 1.35%, and 47.77% higher than that of the other misting treatments T1, T3, and T4, respectively. Under a certain misting duration, the PFP first increased and then decreased with the decrease of micro-spray height, generally showing the pattern of H2 > H1 > H3. The PFP of the middle canopy treatment (H2) was 24.39% and 450.15% higher than that of the upper canopy (H1) and lower canopy (H3) treatments, respectively. The maximum PFP under the interaction of micro-spray height and misting duration was observed in the H2T2 treatment, at 22.39 kg·kg^−1^, indicating that appropriate misting in the middle canopy can generate a higher PFP of fertilizer.

The effects of micro-spray misting on canopy temperature and humidity from 2024 to 2025 are shown in **Figures 9c, d**. After micro-spray misting treatment, canopy temperature decreased, and canopy humidity increased. This effect was more pronounced when appropriate misting was carried out in the middle canopy. However, no significant enhancement in the cooling and humidifying effects was observed under full misting conditions. In the first year of treatment, across a range of misting durations, the average canopy temperature decrease (CTD) generally followed the pattern H2 > H1 > H3. The CTD of the middle canopy treatment (H2) was 9.78% and 22.42% higher than that of the upper canopy (H1) and lower canopy (H3) treatments, respectively. Under a certain misting duration, the average increase in canopy relative humidity (ΔRH) first increased and then decreased with the decrease of micro-spray height, generally showing the pattern of H2 > H1 > H3. The ΔRH of the middle canopy treatment (H2) was 7.80% and 47.65% higher than that of the upper canopy (H1) and lower canopy (H3) treatments, respectively. In 2025, the trends of CTD and ΔRH were consistent with those in 2024. The CTD of the middle canopy treatment (H2) was 11.31% and 79.56% higher than that of the upper canopy (H1) and lower canopy (H3) treatments, respectively. The ΔRH of the middle canopy treatment (H2) was 8.77% and 34.10% higher than that of the upper canopy (H1) and lower canopy (H3) treatments, respectively.

### 3.5 Correlation analysis of different indicators

**Figure 10:**
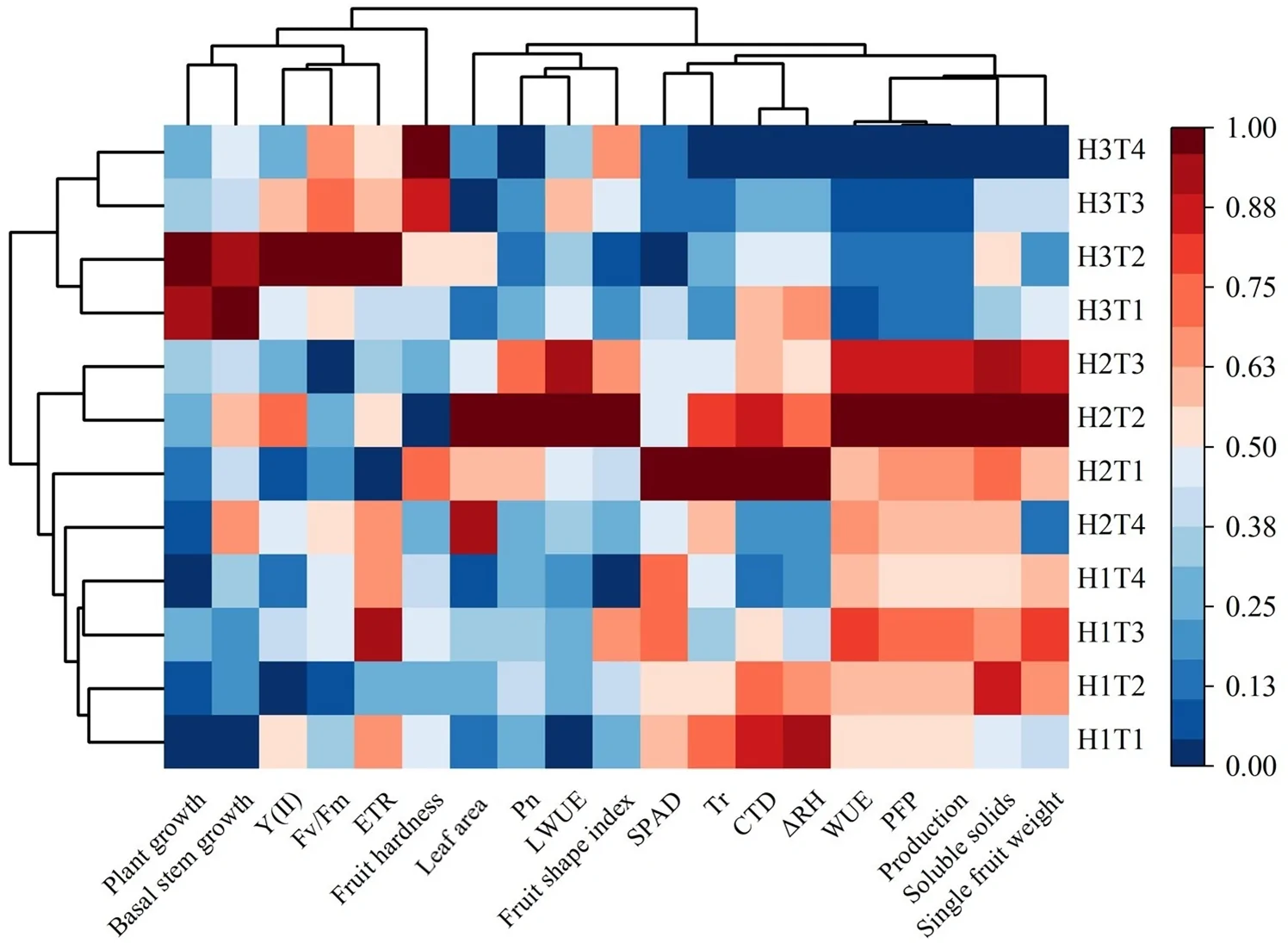
Dendrogram of bidirectional cluster analysis for micro-spray misting treatment.

To characterize the response associations and coordinated variation patterns among apple traits under micro-spray misting treatments, hierarchical clustering heatmaps and Pearson correlation coefficient matrices were constructed. As shown in **Figure 10**, the treatment clusters exhibited distinct response patterns across the measured indicators. The middle-canopy treatments (H2T2 and H2T3) were grouped into Cluster I, characterized by relatively higher values of plant growth, basal stem growth, chlorophyll fluorescence parameters, yield, and fruit quality traits. The upper-canopy treatments (H1T2 and H1T3) formed an intermediate-response cluster, showing relatively stronger responses in vegetative growth and canopy humidity-related indicators. In contrast, the lower-canopy treatments with longer misting durations (H3T3 and H3T4) were classified into Cluster III, exhibiting comparatively weaker responses across most measured indicators. At the indicator level, distinct clustering patterns were also observed. Growth-related traits clustered with chlorophyll fluorescence parameters, suggesting a coordinated variation pattern between vegetative growth and photosynthetic performance, particularly PSII photochemical characteristics. Photosynthetic parameters were closely grouped with water and fertilizer use efficiency, indicating an association between photosynthetic capacity and resource utilization efficiency under micro-spray misting conditions. Furthermore, yield, soluble solids, and single fruit weight were clustered together with fluorescence parameters (Fv/Fm and Y(II)), forming a yield–quality–fluorescence indicator group that showed close correspondence with the high-response treatment cluster. This pattern suggests that improved photosynthetic physiological status was closely associated with enhanced yield and quality responses under suitable misting treatments. Plant growth traits, basal stem growth, and ΔRH were grouped into Cluster II, which corresponded mainly with the intermediate-response treatment cluster. This clustering pattern indicates that upper-canopy treatments were more closely related to vegetative growth responses, whereas their relationships with reproductive development traits were comparatively weaker. Leaf area and fruit hardness formed a relatively independent low-response indicator group, showing limited variation among different spatiotemporal misting treatments. Overall, the clustering and correlation analyses revealed differentiated response patterns among treatments and highlighted the interrelationships among growth, photosynthetic physiology, resource utilization, and yield-quality traits under micro-spray misting regulation.

**Figure 11:**
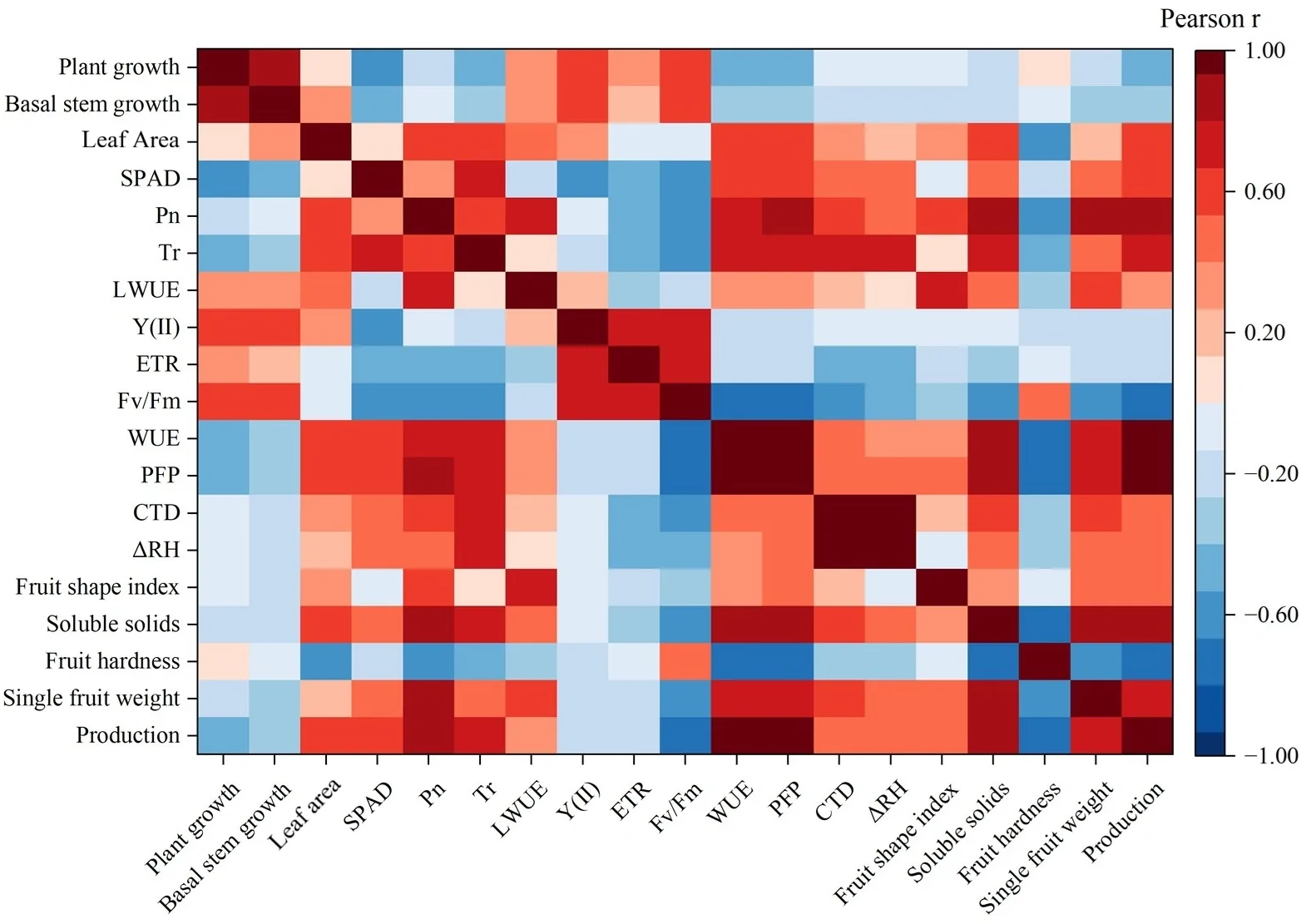
Pearson correlation matrix among various indicators of apples.

**Figure 11** further quantifies the linear associations among different indicators. Plant growth was highly significantly positively correlated with basal stem growth, and both were positively correlated with chlorophyll fluorescence parameters Y(II) and ETR, indicating that improvements in the efficiency of the initial reaction of the photosynthetic system can directly promote growth. Net photosynthetic rate was highly significantly positively correlated with transpiration rate, and both were highly significantly positively correlated with WUE and PFP of fertilizer, reflecting the synergistic optimization effect of photosynthetic efficiency and water and fertilizer resource use efficiency under micro-spray misting treatment. Canopy temperature difference was highly significantly positively correlated with humidity difference, and both were significantly positively correlated with photosynthetic rate and fruit quality indices, indicating that canopy cooling and humidification are the core physiological mechanisms of micro-spray misting regulation. Fruit yield was highly significantly positively correlated with single fruit weight and soluble solids, but negatively correlated with plant growth, reflecting the allocation trade-off between “vegetative growth and reproductive growth” in apple trees. In summary, under micro-spray misting treatment, optimizing the canopy microenvironment can enhance the photosynthetic system’s initial reaction efficiency and photosynthetic rate, thereby driving synergistic improvements in tree growth, water and fertilizer use efficiency, and fruit yield and quality. Furthermore, it also provides a quantitative basis for the subsequent construction of a comprehensive scientific evaluation model.

### 3.6 Comprehensive evaluation model of micro-spray misting

#### 3.6.1 AHP subjective weighting

Based on **Figure 2** and Equations 5–9, the judgment matrices for the criteria layer and each indicator layer were obtained (**Tables 7**–**11**), and the subjective weights for each indicator were calculated (**Table 12**). The consistency test results showed CR< 0.1, indicating that the consistency test was satisfactory and that the established judgment matrices were rational and reliable. Among them, the yield weight was the highest, followed by WUE.

**Table 7:** Criterion layer decision matrix.

Indicator	Growth indicators	Physiological indicators	Resource environment	Yield and quality
Growth Indicators	1	0.333	0.333	0.25
Physiological Indicators	3	1	1	1
Resource Environment	3	1	1	1
Yield and quality	4	1	1	1

**Table 8:** Growth index layer decision matrix.

Indicator	Plant growth	Basal stem growth	Leaf area
Plant growth	1	3	2
Basal stem growth	0.333	1	1
LA	0.5	1	1

**Table 9:** Physiological indicator layer decision matrix.

Indicator	SPAD	Pn	Tr	LWUE	Y(II)	ETR	Fv/Fm
SPAD	1	0.2	0.333	0.143	0.167	0.167	0.25
Pn	5	1	1	1	1	1	1
Tr	3	1	1	1	1	1	1
LWUE	7	1	1	1	1	1	1
Y(II)	6	1	1	1	1	1	1
ETR	6	1	1	1	1	1	1
Fv/Fm	4	1	1	1	1	1	1

**Table 10:** Resource and environmental indicator layer decision matrix.

Indicator	WUE	PFP	CTD	ΔRH
WUE	1	3	2	5
PFP	0.333	1	1	1
CTD	0.5	1	1	1
ΔRH	0.2	1	1	1

**Table 11:** Yield and quality indicator layer decision matrix.

Indicator	Fruit shape index	Soluble solids	Fruit hardness	Single fruit weight	Production
Fruit shape index	1	0.2	0.25	0.143	0.125
Soluble solids	5	1	1	1	1
Fruit hardness	4	1	1	1	1
Single fruit weight	7	1	1	1	1
Production	8	1	1	1	1

**Table 12:** AHP subjective weight calculation results.

Indicator ID	Indicator name	Pertaining guideline	Guideline weight	Indicator layer weight	Composite weight	Ranking	Consistency test
C1	Plant growth	Growth indicators	0.092	0.549	0.050	8	CI=0.009 RI=0.525 CR=0.017<0.1
C2	Basal stem growth	Growth indicators	0.092	0.211	0.019	17	
C3	Leaf area	Growth indicators	0.092	0.241	0.022	15	
C4	SPAD	Physiological indicators	0.295	0.033	0.010	19	CI=0.010 RI=1.341 CR=0.008<0.1
C5	Pn	Physiological indicators	0.295	0.160	0.047	9	
C6	Tr	Physiological indicators	0.295	0.151	0.045	10	
C7	LWUE	Physiological indicators	0.295	0.169	0.050	7	
C8	Y(II)	Physiological indicators	0.295	0.165	0.049	11	
C9	ETR	Physiological indicators	0.295	0.165	0.049	11	
C10	Fv/Fm	Physiological indicators	0.295	0.156	0.046	13	
C11	WUE	Resource environment	0.295	0.504	0.149	2	CI=0.027 RI=0.882 CR=0.030<0.1
C12	PFP	Resource environment	0.295	0.164	0.048	12	
C13	CTD	Resource environment	0.295	0.184	0.054	6	
C14	ΔRH	Resource environment	0.295	0.148	0.044	14	
C15	Fruit shape index	Yield and quality	0.318	0.042	0.013	18	CI=0.012 RI=1.110 CR=0.011<0.1
C16	Soluble solids	Yield and quality	0.318	0.231	0.074	5	
C17	Fruit hardness	Yield and quality	0.318	0.223	0.071	4	
C18	Single fruit weight	Yield and quality	0.318	0.247	0.079	3	
C19	Production	Yield and quality	0.318	0.255	0.081	1	

#### 3.6.2 Objective weighting of CV

Using Equations 10–12, objective weights were calculated via the CV method. The results are shown in **Table 13**, where WUE carries the highest weight, followed by yield, while the fruit shape index has the lowest weight.

**Table 13:** Objective weight calculation results.

Indicator	Mean	Standard deviation	CV	Weight (%)
Plant growth	52.523	20.490	0.390	9.551
Fv/Fm	0.750	0.028	0.037	0.905
Basal stem growth	11.047	2.593	0.235	5.746
LA	0.612	0.118	0.193	4.724
PFP	11.817	6.948	0.588	14.396
Y(II)	0.601	0.015	0.025	0.613
Tr	2.473	0.184	0.075	1.825
ΔRH	4.546	1.620	0.356	8.724
**WUE**	**3.029**	**1.784**	**0.589**	**14.418**
Fruit hardness	8.038	0.306	0.038	0.932
CTD	3.208	1.127	0.351	8.604
Fruit shape index	0.802	0.018	0.023	0.555
Soluble solids	15.223	0.411	0.027	0.661
Production	2904.284	1707.891	0.588	14.398
Single fruit weight	120.530	14.981	0.124	3.043
ETR	11.212	0.518	0.046	1.132
SPAD	52.953	2.720	0.051	1.258
LWUE	4.236	0.621	0.147	3.590
Pn	9.964	2.005	0.201	4.928

Bold values indicate the indicator with the highest objective weight.

#### 3.6.3 GT combined weighting

Based on the above subjective and objective weights, the combined weights were calculated using GT through Equations 13–16, and the results are shown in **Table 14**. The final combined weights of the indicators, in descending order, are as follows: WUE > Yield > PFP > Plant growth > CTD > ΔRH > Basal stem growth > Pn > Leaf area > LWUE > Single fruit weight > Tr > Firmness > ETR > Soluble solids > Fv/Fm > Y(II) > SPAD > Fruit shape index. Among them, WUE, yield, and PFP rank among the top three, which is highly consistent with the goal of efficient water resource utilization. This indicates that the weight allocation aligns with the study’s scientific issues.

**Table 14:** Game theory combinatorial weighting calculation results.

Indicator	Subjective weight	Objective weight	Combined weight	Ranking
**WUE**	**0.149**	**0.144**	**0.145**	**1**
Production	0.081	0.144	0.134	2
Single fruit weight	0.079	0.030	0.038	11
Fruit hardness	0.071	0.009	0.019	13
Plant growth	0.050	0.096	0.088	4
Soluble solids	0.074	0.007	0.017	15
CTD	0.054	0.086	0.081	5
Pn	0.047	0.049	0.049	8
Y(II)	0.049	0.006	0.013	17
LWUE	0.050	0.036	0.038	10
PFP	0.048	0.144	0.129	3
ΔRH	0.044	0.087	0.080	6
Fv/Fm	0.046	0.009	0.015	16
Tr	0.045	0.018	0.022	12
ETR	0.049	0.011	0.017	14
Leaf area	0.022	0.047	0.043	9
Basal stem growth	0.019	0.057	0.051	7
Fruit shape index	0.013	0.006	0.007	19
SPAD	0.010	0.013	0.012	18

Bold values indicate the indicator ranked first according to the combined weight.

#### 3.6.4 AHP-CV-RSR comprehensive evaluation model

**Figure 12:**
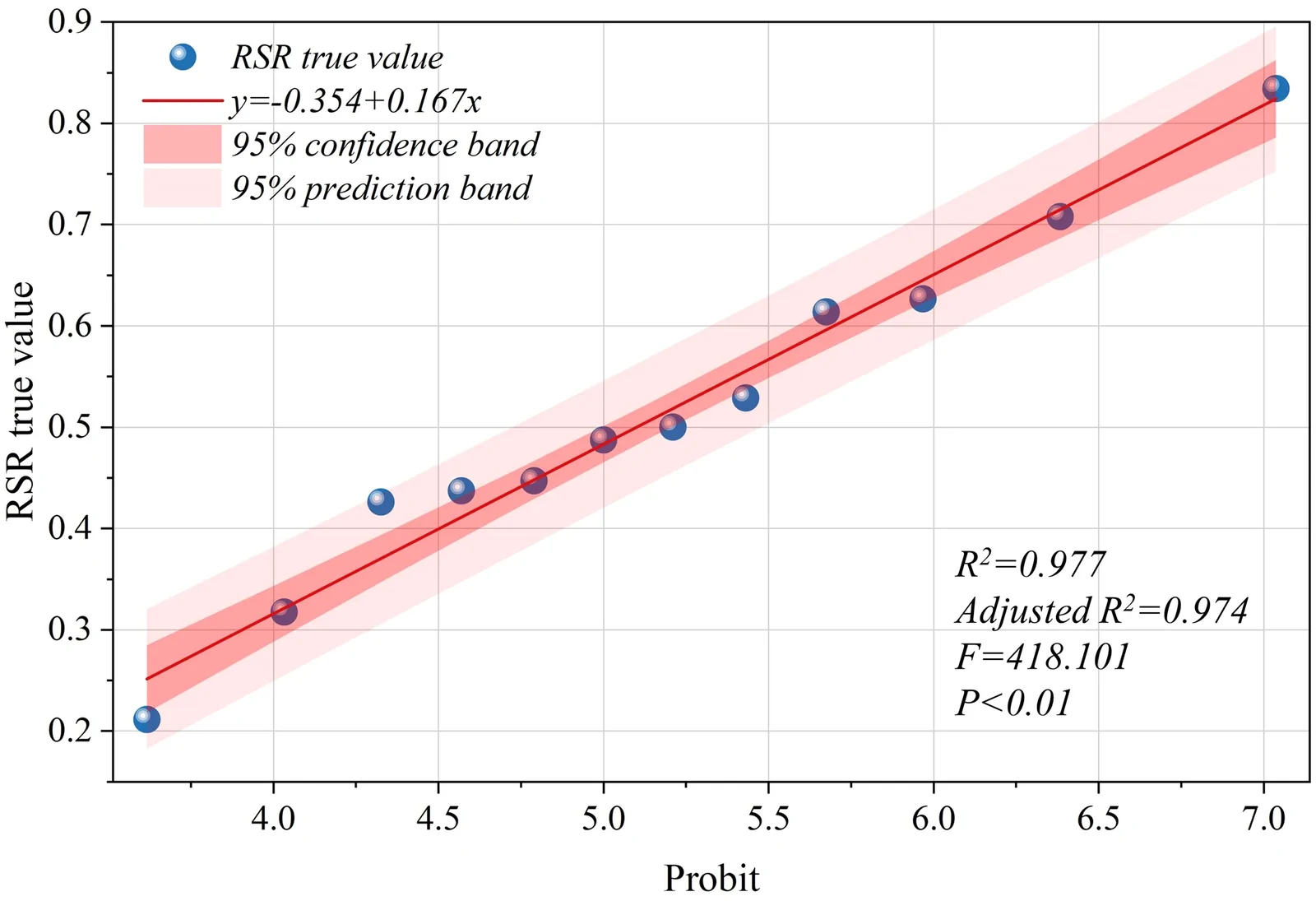
Fitting effect diagram.

The combined weights were substituted into the RSR comprehensive evaluation model, and the RSR frequency distribution was obtained as shown in **Table 15**. A linear regression analysis was conducted with the probit value as the independent variable and RSR as the dependent variable, resulting in R² = 0.977. The fitting effect is shown in **Figure 12**, indicating that the model performed well. Then, the RSR estimates calculated from the regression equation were used for ranking and classification (**Table 16**). Among them, the H2T2 and H2T3 treatments were classified as the best category, and the RSR fitting value for the H2T2 treatment (0.824) was significantly higher than those of the other treatments, ranking first. Moreover, it indicates that the H2T2 treatment achieved the best balance in improving canopy microclimate, promoting tree physiological metabolism, and enhancing fruit quality, with the optimal comprehensive benefits.

**Table 15:** RSR distribution table.

RSR	Frequency	Σf	Evaluation rank	Evaluation rank/n*100%	Probit
0.211	1	1	1	8.333	3.617
0.318	1	2	2	16.667	4.033
0.426	1	3	3	25.000	4.326
0.437	1	4	4	33.333	4.569
0.447	1	5	5	41.667	4.790
0.488	1	6	6	50.000	5.000
0.500	1	7	7	58.333	5.210
0.529	1	8	8	66.667	5.431
0.614	1	9	9	75.000	5.674
0.627	1	10	10	83.333	5.967
0.708	1	11	11	91.667	6.383
0.834	1	12	12	97.917	7.037

**Table 16:** Graded classification results.

Index	RSR rank	Probit	RSR fitted value	Classification level
**H2T2**	**1**	**7.037**	**0.824**	**3**
H2T3	2	6.383	0.715	3
H1T1	7	5.000	0.483	2
H1T2	5	5.431	0.555	2
H1T3	4	5.674	0.596	2
H1T4	10	4.326	0.370	2
H2T1	3	5.967	0.645	2
H2T4	6	5.210	0.518	2
H3T1	9	4.569	0.411	2
H3T2	8	4.790	0.448	2
H3T3	11	4.033	0.321	2
H3T4	12	3.617	0.252	1

Bold values indicate the treatment ranked first according to the fitted RSR value.

### 3.7 Response surface optimization of misting height and duration

To further identify the optimal combination of misting height and misting duration, a quadratic response surface model was fitted using coded variables. The model exhibited strong explanatory power, with an R^2^ of 0.9443 and an adjusted R^2^ of 0.8979, indicating that the fitted surface captured most of the variation in the integrated score. The overall model was highly significant (F = 20.3573, p = 0.0011). Among the model terms listed in **Table 17**, the linear effects of misting height (p = 0.009) and misting duration (p = 0.015) were both significant, as were the quadratic terms for misting height (p< 0.001) and misting duration (p = 0.005). In contrast, the interaction term was not significant (p = 0.398), suggesting that the integrated response was driven primarily by the individual nonlinear effects of the two factors rather than by a strong height-by-duration interaction. The low condition number (5.27) further indicated that multicollinearity was not a concern in the coded-variable model.

**Table 17:** Regression coefficients and statistical significance of the quadratic response surface model describing the integrated response to misting height and misting duration.

Term	Coefficient	P-value
Intercept	0.7568	<0.001
H	-0.0715	0.009
T	-0.0464	0.015
H²	-0.2460	<0.001
H × T	-0.0153	0.398
T²	-0.0650	0.005

**Figure 13:**
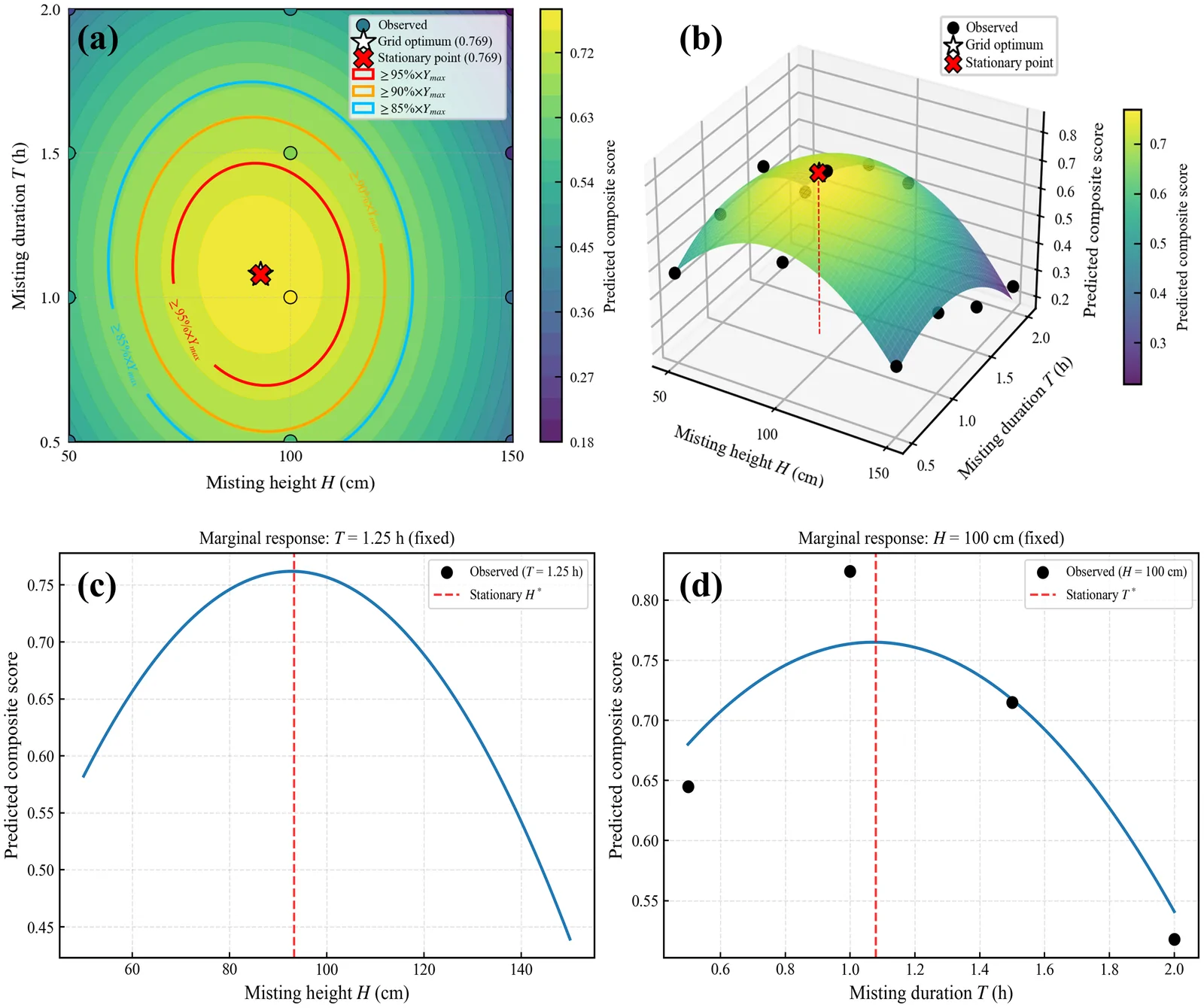
Quadratic response surface analysis of integrated performance as affected by misting height and duration. **(a)** Two-dimensional contour plot showing observed treatments, the stationary point, and feasible high-performance regions. **(b)** Three-dimensional response surface with the predicted optimum and stationary point. **(c, d)** Marginal response curves illustrating the nonlinear effects of misting height and duration when the other factor was fixed at its central level.

The fitted surface revealed a clear internal maximum within the experimental domain (**Figures 13a, b**). The stationary point was located at a misting height of 93.26 cm and a misting duration of 1.08 h, with a predicted RSR comprehensive evaluation score of 0.7695. The fitted quadratic regression equation in coded variables is: $Y=0.7568-0.0715X_{\text{H}}-0.0464X_{\text{T}}-0.2460X_{{\text{H}}^{2}}-0.0650X_{{\text{T}}^{2}}-0.0153X_{\text{H}}X_{\text{T}}$, where Y is the predicted RSR score, *X_H_* is the coded variable for misting height ranging from -1.0 to +1.0 (corresponding to physical heights of 50 to 150 cm, coded as XH = (H - 100)/50), and *X_T_* is the coded variable for misting duration ranging from -1.5 to +1.5 (corresponding to physical durations of 0.5 to 2.0 h, coded as XT = (T - 1.25)/0.5). The Hessian matrix had two negative eigenvalues (−0.492646 and −0.129354), confirming that the stationary point corresponded to a local maximum rather than a minimum or saddle point. Importantly, as illustrated by the stationary-point location in **Figures 13a, b**, the optimum fell within the experimental range, suggesting that the identified optimum was supported by the observed design space rather than being a pure extrapolation.

Although the highest observed score was obtained under the H2T2 treatment (0.824), the response surface in **Figures 13a, b** provided a smoothed representation of the overall trend and therefore yielded a slightly lower theoretical optimum. This difference is expected because quadratic fitting emphasizes the global response pattern rather than isolated local peaks. In practical terms, the response surface suggested that moderate misting height combined with intermediate misting duration formed the center of a stable high-performance region.

The high-performance operating window was further quantified by threshold analysis based on a proportion of the predicted maximum response, as visualized by the feasible regions in **Figure 13a**. A region exceeding 95% of the maximum predicted score (≥ 0.7310) was identified within H = 73.65–112.93 cm and T = 0.70–1.46 h. When the threshold was relaxed to 90% of the maximum predicted score (≥ 0.6925), the feasible region expanded to H = 65.43–121.14 cm and T = 0.54–1.62 h. A broader but still connected feasible zone was obtained at 85% of the maximum predicted score (≥ 0.6541), spanning H = 59.02–127.56 cm and T = 0.50–1.74 h. All three thresholds formed single connected regions, indicating that the response surface contained a compact and stable high-performance domain rather than multiple disconnected optima.

The marginal response curves in **Figures 13c, d** provided additional support for these results. When misting duration was held constant at the central level, the predicted score increased initially with misting height and then declined after reaching the optimum region, indicating a clear concave relationship (**Figure 13c**). A similar unimodal pattern was observed for misting duration when misting height was fixed at its central level (**Figure 13d**). Together, these curves confirmed that both excessive and insufficient settings reduced the integrated performance, whereas intermediate values of the two factors favored the best response.

## 4 Discussion

### 4.1 Effects of micro-spray misting on apple growth, development, and fruit quality

The growth and development of fruit trees and fruit quality serve as core indicators for evaluating the regulatory effects of cultivation techniques. The growth and development process determines the accumulation of photosynthetic products and the tree’s potential for stress resistance, while fruit quality directly impacts the fruit’s commercial value and market competitiveness (Koyro and Huchzermeyer, 2018; He et al., 2022). The synergistic improvement of both is key to improving the quality and efficiency of orchards (Zhang et al., 2024). In this study, the growth of apple trees in the second year of treatment was generally higher than in the first year, consistent with the growth characteristics of fruit trees. As the tree ages, the root system expands, enhancing the ability to accumulate substances (Jia et al., 2025). It was found that in the second year of treatment (2025), micro-spray misting had no significant effect on overall basal stem growth, whereas treatment H3 showed greater growth compared to other heights. This result may be related to a shift in the tree’s growth focus during its growth process. As the tree matures and undergoes ontogenetic shifts, the basal stem progressively becomes lignified, reducing its sensitivity to distal canopy microenvironment regulation (i.e., from H1 and H2 settings) (Cancino et al., 2026). Concurrently, however, the growth demands of the root system and lower scaffold branches increase during the transition from juvenile vegetative vigor to early reproductive maturity. This shift aligns the microclimate-modifying effects of localized lower canopy spraying (H3) with the localized resource allocation priorities of the basal portion of the tree (Yang et al., 2025)—offering direct microclimate protection at the stem base that was not yet prominent during the purely vegetative phase in 2024. In both years, leaf area was optimal for the middle-canopy treatment (H2). In the first year, it was 48.39% and 51.65% higher than the upper canopy (H1) and lower canopy (H3) treatments, respectively, and in the second year, it was 28.20% and 23.27% higher, respectively. It may be because micro-spray misting in the middle canopy can effectively alleviate the inhibition of leaf cell division caused by high temperature and drought, promoting leaf growth and development (Luan and Vico, 2021; Chen et al., 2023). The response patterns of fruit yield and quality indicate that the micro-spray location is the primary factor in regulating fruit yield and quality, suggesting that spatial micro-spray regulation has a greater impact on fruit. An appropriate micro-spray height can optimize the tree’s photosynthesis and nutrient transport processes, thereby improving yield and quality (Zhang et al., 2022). Among them, the fruit of the H2T2 treatment had the best comprehensive performance, indicating that an appropriate amount of misting in the middle canopy not only met the water needs of fruit development but also did not cause excessive canopy humidity due to full misting, which would affect sugar accumulation in the fruit (Pieper et al., 2024). There were no significant differences in fruit shape index among different treatments. The analysis suggests that fruit shape is mainly determined by the genetic characteristics of the variety, which is consistent with the conclusions of Dujak et al. (2024), and external regulation has a limited impact on fruit shape. The above results show that appropriate micro-spray misting can promote leaf growth and development, enhance the tree’s ability to accumulate photosynthetic products, and precisely match water needs during the fruit development period, thereby improving apple quality and enhancing efficiency (Khot et al., 2012).

### 4.2 Regulatory mechanisms of micro-spray misting on apple physiological characteristics

Under high temperature and drought stress, researching apple physiological characteristics is crucial for revealing their stress response mechanisms (Wang et al., 2024). Chlorophyll content, photosynthetic characteristics, and fluorescence parameters, as key indicators of fruit tree physiological status, can not only indicate carbon assimilation capacity and water-use efficiency but also reflect the operation state of PSII and the degree of photoinhibition (Zhou et al., 2023). In this study, the SPAD value of apples showed a pattern of “first increasing, then decreasing, and finally stabilizing” with the growth stage, consistent with the results of Zheng et al. (2025) on drip-irrigated apples. The middle canopy treatment (H2) maintained a higher SPAD value in both years, possibly because micro-spray in the middle canopy can effectively avoid the inhibition of chlorophyll synthase activity by high temperature and drought, while also reducing the chlorophyll degradation rate, providing a material basis for photosynthesis (Zhao et al., 2025). In addition, the SPAD value and Pn synchronously decreased slightly in the second year, which may be due to the gradual shift of the growth focus from “vegetative growth” to “reproductive growth” (Verma and Bodh, 2025). Alternative explanations include potential nutrient dilution associated with the heavier fruit load in 2025 and natural leaf aging as the trees mature, both of which may have contributed to the slight decline in SPAD values observed during the second year. This interannual consistency between SPAD and Pn dynamics further confirms the direct regulatory role of chlorophyll content on light capture and carbon assimilation efficiency (Li et al., 2025). In 2025, the indicators Y(II), ETR, and Fv/Fm were generally higher than in 2024, indicating that micro-spray misting has a significant interannual cumulative effect on fluorescence parameters. Long-term appropriate micro-spray treatment can effectively protect the leaf photosynthetic apparatus from high-temperature and drought-stress damage, thereby improving the efficiency and stability of the light reaction process. Similar physiological responses have also been reported in tomato plants under micro-spray misting (Xue et al., 2025), although direct comparisons between species should be interpreted with caution. It is confirmed that under high temperature and drought conditions, micro-spray misting can optimize the canopy microenvironment of apple trees, protect the structure of the photosynthetic reaction center, maintain the efficiency of photosynthetic electron transfer and photosystem energy conversion, and thus effectively enhance the photosynthetic physiological stress resistance of apple trees against high temperature and drought stress (Weng et al., 2022).

### 4.3 Regulation of resource use efficiency and orchard microclimate by micro-spray misting

The orchard microclimate directly determines the potential to improve quality and enhance efficiency, as well as the sustainability of orchard production (Abasi et al., 2025). Scientific regulation of canopy microclimate and improvement of water and fertilizer use efficiency are not only key pathways to optimizing apple cultivation physiological processes but also important supports for promoting the green and sustainable development of the fruit tree industry (Zhang et al., 2019; Ali et al., 2024). This study found that micro-spray misting can significantly reduce apple canopy temperature and increase relative humidity. With increased misting duration, the cooling and humidifying effects became more pronounced. However, when the duration increased from 1.5 h to 2.0 h, there was no significant improvement in the cooling and humidifying effects. It may be because a certain amount of misting water enables the canopy microenvironment to reach the regulatory threshold. Full misting increases water consumption but does not significantly further optimize the canopy microclimate, resulting in relatively weak regulatory benefits (Zheng et al., 2021). In addition, as micro-spray height increased, the cooling and humidifying effects first increased and then decreased. The middle canopy had a significant cooling and humidifying effect. It may be because the middle canopy is the concentrated distribution area of branches and leaves, which can avoid the mist droplets from the upper canopy misting evaporating quickly and the lower canopy misting being blocked by the canopy and falling on the ground (Hu and Whitty, 2019), which is consistent with the research patterns of Wünsche et al. (2004) on apples. Related studies have shown that canopy microclimate can significantly affect water and fertilizer use efficiency and thus affect fruit quality (Ou et al., 2023), which is consistent with the conclusion of this study that the middle canopy has higher water and fertilizer use efficiency. In this study, the WUE and PFP of fertilizer under the H2T2 treatment were the highest, which were 54.35% and 51.28% higher than those of the H2T1 treatment at the same height, respectively. The reason may be that the H2T1 treatment had excessive misting, leading to water waste and soil nutrient leaching, ultimately decreasing water and fertilizer use efficiency (Nikolskii and Aidarov, 2023). From a practical perspective, the 1.5 h misting duration (H2T2) achieves the best balance between water and energy input costs and yield/quality gains. The 1.0 h regime (H2T3), classified in the same top-performance tier, represents a viable water-saving alternative with marginally lower but still favorable comprehensive performance and may be preferred in regions with more severe water scarcity or higher energy costs.

### 4.4 Construction and application of the comprehensive evaluation model

Comprehensive evaluation is a key step in assessing the impact of irrigation patterns on the growth and development of fruit trees, providing a scientific basis for the joint optimization of physiological growth indices, yield formation, and resource use efficiency (He et al., 2021a). Its core value lies in systematically integrating multi-dimensional indicators to provide a quantitative basis for decision-making on the joint optimization goals of irrigation patterns, thereby avoiding the one-sidedness of single-indicator evaluation (Tong et al., 2022). This study, targeting the comprehensive benefits of orchard micro-spray misting regulation, adopted a combined “subjective + objective” weighting strategy. The AHP was used to complete the subjective weighting. By constructing a judgment matrix and conducting a consistency test, the CR values for all criteria and indicator levels were less than 0.1, indicating that the judgment matrix was constructed reasonably and that the weight allocation had a reliable, logical basis (Donati et al., 2023). The CV method was then used for objective weighting. The results showed that WUE had the highest weight, followed by yield, indicating that these two indicators were the most sensitive to the spatiotemporal treatments of micro-spray misting. The GT combined weight ranking showed that WUE, yield, and PFP were the top three, which was consistent with the dual goals of this study of “quality improvement and efficiency enhancement + water-saving and high efficiency,” fully demonstrating the high scientific and rational nature of the GT combined weight allocation (Ganjali and Guney, 2025). Notably, the response surface analysis showed that the interaction term of misting height and misting duration (H×T) was not statistically significant (p = 0.398). The optimal parameter combination identified in this study reflects the joint optimization of both factors at their intermediate levels, rather than a synergistic statistical interaction effect between the two factors. The final AHP-CV-RSR comprehensive evaluation model results showed that the RSR fitting value for the H2T2 treatment was 0.824, significantly higher than those of all other treatments and ranking first. This result was highly consistent with the conclusions of the single-indicator analysis in the previous section. The model also divided the different treatments into three levels, clearly distinguishing the comprehensive regulatory benefits of each spatiotemporal configuration. This method provides a clear quantitative basis for selecting the appropriate mode according to actual needs in production practice. Among them, the H2T2 and H2T3 treatments were in the best category. In water-scarce areas, the H2T3 treatment can be considered for regulation.

### 4.5 Implications of response surface optimization for orchard microclimate regulation

The response surface analysis extended the treatment-level comparison by transforming discrete experimental observations into a continuous optimization framework, thereby identifying both the stationary optimum and the broader high-performance operational domain (Emran et al., 2024). Although the H2T2 treatment produced the highest observed integrated score, the fitted response surface yielded a slightly lower stationary optimum, reflecting the smoothing nature of quadratic approximation and its emphasis on overall response trends rather than isolated local peaks. Importantly, the stationary point was located within the experimental domain, indicating that the predicted optimum was supported by the observed data structure rather than mathematical extrapolation. The identified response pattern suggests that moderate misting height combined with intermediate misting duration provides the most favorable balance between canopy cooling efficiency and excessive moisture accumulation. Excessively low misting height may restrict droplet distribution within the canopy, whereas overly high placement likely increases droplet drift and evaporation loss before effective canopy penetration. Similarly, insufficient misting duration may limit evaporative cooling, while prolonged misting may elevate local humidity and weaken transpiration-driven cooling efficiency. Collectively, these findings indicate that both insufficient and excessive misting intensity can constrain eco-physiological performance, whereas intermediate operating conditions maximize canopy cooling and microenvironment stabilization.

From a practical perspective, the response surface analysis suggests that orchard management should prioritize stable operational ranges rather than a single exact optimum. The connected high-performance regions identified under different threshold levels indicate that the system possesses relatively strong tolerance to moderate fluctuations in misting height and duration, which is particularly important under field conditions where precise parameter control is difficult to maintain. Therefore, the practical value of the model lies not only in identifying an optimal parameter combination but also in defining a robust management window capable of sustaining favorable physiological responses under realistic orchard conditions.

While this study provides new insights into the spatiotemporal regulation of micro-spray misting in apple orchards, several limitations should be acknowledged. The experiment was conducted using a single apple cultivar (‘Yanfu No. 8’) under semi-arid conditions in the Central Plains of China, and the applicability of the proposed misting strategy across different cultivars, orchard systems, and climatic conditions therefore requires further evaluation. In addition, the study was conducted over a relatively limited temporal scale, and long-term multi-season observations are needed to determine whether the identified optimal operational ranges remain stable under interannual climatic variability and prolonged heat stress conditions. Furthermore, the present study primarily focused on integrated eco-physiological responses and water-use characteristics. Direct measurements of stomatal conductance and leaf water potential were not included due to equipment constraints, which limited the ability to fully elucidate the mechanistic pathways (e.g., reduced stomatal limitation *vs*. direct cooling effects) underlying the observed improvements in photosynthetic performance. Future studies incorporating stomatal conductance, leaf water potential, continuous canopy microclimate monitoring, and transpiration measurements across multiple cultivars and regions would help clarify how micro-spray misting dynamically influences canopy cooling, plant water relations, and resource-use efficiency, and would further strengthen the robustness and field applicability of the proposed misting strategies. Addressing these aspects would further strengthen the robustness and field applicability of micro-spray regulation strategies for sustainable orchard water management under future climate change scenarios.

## 5 Conclusion

This study demonstrated that the responses of semi-arid apple orchards to micro-spray misting depend on both misting height and operating duration. Among the tested treatments, moderate misting applied within the middle canopy for 1.5 h (H2T2) provided the most favorable overall orchard performance under the experimental conditions, improving canopy microclimate regulation, physiological stability, fruit production, and water-use efficiency. Response surface analysis further identified a relatively stable high-performance operational region within the tested parameter range, indicating that practical orchard management should prioritize feasible operational ranges rather than relying solely on isolated treatment optima. Overall, these findings suggest that spatiotemporally optimized micro-spray misting provides a practical approach for improving orchard microclimate regulation and sustaining orchard performance under heat and drought stress in semi-arid regions.

## Data Availability

The datasets generated and analyzed during the current study are available from the corresponding author upon reasonable request.

## References

[B1] Abasi F. Hussain S. Mashwani Z.-R. Raja N. I. (2025). “ Orchards management under changing climate,” in Challenges and Solutions of Climate Impact on Agriculture (London: Elsevier) 145–162. doi: 10.1016/B978-0-443-23707-2.00006-4

[B2] Ali N. Dong Y. Lavely E. (2024). Impact of irrigation scheduling on yield and water use efficiency of apples, peaches, and sweet cherries: A global meta-analysis. Agric. Water Manage. 306, 109148. doi: 10.1016/j.agwat.2024.109148 38826717

[B3] Blanco V. Kalcsits L. (2024). Relating microtensiometer-based trunk water potential with sap flow, canopy temperature, and trunk and fruit diameter variations for irrigated ‘Honeycrisp’ apple. Front. Plant Sci. 15. doi: 10.3389/fpls.2024.1393028 38855474 PMC11157117

[B4] Cai S. Zheng B. Zhao Z. Zheng Z. Yang N. Zhai B. (2023). Precision nitrogen fertilizer and irrigation management for apple cultivation based on a multilevel comprehensive evaluation method of yield, quality, and profit indices. Water 15, 468. doi: 10.3390/w15030468 30654563

[B5] Cancino J. Acuña E. Sandoval S. Espinosa M. (2026). Comparative study of sapwood volume as an indicator of biological rotation age in six varieties of poplar. For. Int. J. For. Res. 99, cpaf026. doi: 10.1093/forestry/cpaf026

[B6] Chandel A. K. Khot L. R. Stöckle C. O. Kalcsits L. Mantle S. Rathnayake A. P. . (2025). Canopy transpiration mapping in an apple orchard using high-resolution airborne spectral and thermal imagery with weather data. AgriEngineering 7, 154. doi: 10.3390/agriengineering7050154 30654563

[B7] Chen M. Zhang T.-L. Hu C.-G. Zhang J.-Z. (2023). The role of drought and temperature stress in the regulation of flowering time in annuals and perennials. Agronomy 13, 3034. doi: 10.3390/agronomy13123034 30654563

[B8] Dag A. Vitoshkin H. Resef G. Ron Y. Alchanatis V. (2026). Evaluating overhead sprinklers and sprayers for heatwave protection in avocado orchards. Plants 15, 1516. doi: 10.3390/plants15101516 42197649 PMC13210729

[B9] Devin S. R. Prudencio Á.S. Mahdavi S. M. E. Rubio M. Martínez-García P. J. Martínez-Gómez P. (2023). Orchard management and incorporation of biochemical and molecular strategies for improving drought tolerance in fruit tree crops. Plants 12, 773. doi: 10.3390/plants12040773 36840120 PMC9960531

[B10] Donati I. I. M. Viaggi D. Srdjevic Z. Srdjevic B. Di Fonzo A. Del Giudice T. . (2023). An analysis of preference weights and setting priorities by irrigation advisory services users based on the analytic hierarchy process. Agriculture 13, 1545. doi: 10.3390/agriculture13081545 30654563

[B11] Dujak C. Coleto-Alcudia V. Aranzana M. J. (2024). Genomic analysis of fruit size and shape traits in apple: unveiling candidate genes through GWAS analysis. Hortic. Res. 11, uhad270. doi: 10.1093/hr/uhad270 38419968 PMC10901474

[B12] Emran M. El-Gamal E. H. Haddad A. M. Ibrahim O. M. (2024). Response surface methodology for optimizing water and fertilizer requirements for maize (Zea mays L.) growth in sandy soil. J. Soil Sci. Plant Nutr. 24, 6349–6364. doi: 10.1007/s42729-024-01973-w 30311153

[B13] Ganjali N. Guney C. (2025). Balancing economic and environmental objectives in agriculture: A game theory analysis. Process. Integr. Optim. Sustain. 9, 1445–1461. doi: 10.1007/s41660-025-00517-8 30311153

[B14] Hao K. Fei L. Liu L. Jie F. Peng Y. Liu X. . (2022). Comprehensive evaluation on the yield, quality, and water-nitrogen use efficiency of mountain apple under surge-root irrigation in the Loess Plateau based on the improved TOPSIS method. Front. Plant Sci. 13. doi: 10.3389/fpls.2022.853546 35449894 PMC9018000

[B15] Hao S. Cao H. Wang H. Pan X. (2019). Effects of water stress at different growth stages on comprehensive fruit quality and yield in different bunches of tomatoes in greenhouses. Int. J. Agric. Biol. Eng. 12, 67–76. doi: 10.25165/j.ijabe.20191203.4468

[B16] He L. Fang W. Zhao G. Wu Z. Fu L. Li R. . (2022). Fruit yield prediction and estimation in orchards: A state-of-the-art comprehensive review for both direct and indirect methods. Comput. Electron. Agric. 195, 106812. doi: 10.1016/j.compag.2022.106812 38826717

[B17] He Z. Hong T. Cai Z. Yang Z. Li M. Zhang Z. (2021a). Determination of amount of irrigation and nitrogen for comprehensive growth of greenhouse cucumber based on multi-level fuzzy evaluation. Int. J. Agric. Biol. Eng. 14, 35–42. doi: 10.25165/j.ijabe.20211402.5785

[B18] He Z. Li M. Cai Z. Zhao R. Hong T. Yang Z. . (2021b). Optimal irrigation and fertilizer amounts based on multi-level fuzzy comprehensive evaluation of yield, growth and fruit quality on cherry tomato. Agric. Water Manage. 243, 106360. doi: 10.1016/j.agwat.2020.106360 38826717

[B19] Herpin S. Mballo S. Manteau M. Lemesle D. Boukouya A. Dubuc B. . (2024). A reduced-scale canyon street to study tree climate benefits: summer 2020 data with well-watered apple trees. Sci. Data 11, 1015. doi: 10.1038/s41597-024-03650-0 39294160 PMC11411096

[B20] Hu M. Whitty M. (2019). An evaluation of an apple canopy density mapping system for a variable-rate sprayer. IFAC-Pap. 52, 342–348. doi: 10.1016/j.ifacol.2019.12.563 38826717

[B21] Jia X. Xu S. Wei J. Wang F. Qing Y. Zhang Z. . (2025). Cytokinin mediates age‐dependent drought response by regulating the removal reactive oxygen species in apple. Plant J. 121, e70023. doi: 10.1111/tpj.70023 39994960

[B22] Khot L. R. Ehsani R. Albrigo G. Larbi P. A. Landers A. Campoy J. . (2012). Air-assisted sprayer adapted for precision horticulture: Spray patterns and deposition assessments in small-sized citrus canopies. Biosyst. Eng. 113, 76–85. doi: 10.1016/j.biosystemseng.2012.06.008 38826717

[B23] Koyro H.-W. Huchzermeyer B. (2018). “ Coordinated regulation of photosynthesis in plants increases yield and resistance to different types of environmental stress,” in Plant Metabolites and Regulation Under Environmental Stress (London: Elsevier) 281–309. doi: 10.1016/B978-0-12-812689-9.00014-5

[B24] Kumar S. Chatterjee U. David Raj A. Sooryamol K. R. (2023). “ Global warming and climate crisis/extreme events,” in Climate Crisis: Adaptive Approaches and Sustainability. Eds. Chatterjee U. Shaw R. Kumar S. D. Raj A. Das S. ( Springer Nature Switzerland, Cham), 3–18. doi: 10.1007/978-3-031-44397-8_1

[B25] Li X. Li S. Qiang X. Yu Z. Sun Z. Wang R. . (2024). Effects of water and nitrogen regulation on apple tree growth, yield, quality, and their water and nitrogen utilization efficiency. Plants 13, 2404. doi: 10.3390/plants13172404 39273888 PMC11396910

[B26] Li Q. Liu Z. Yang Y. Han Y. Wang X. (2023). Evaluation of water resources carrying capacity in Tarim River Basin under game theory combination weights. Ecol. Indic. 154, 110609. doi: 10.1016/j.ecolind.2023.110609 38826717

[B27] Li L.-X. Yang S.-K. Fang Y. Wu Z.-M. Ma H.-Y. Wang S. . (2025). Transcription factors MdEIL1 and MdHY5 integrate ethylene and light signaling to promote chlorophyll degradation in mature apple peels. Hortic. Res. 12, uhae324. doi: 10.1093/hr/uhae324 40236981 PMC11997652

[B28] Liu H. Bai Y. Zhang J. Zheng M. Ding P. (2020). Effects of microclimate factors on grape fruit growth and sugar content in extreme arid regions under different micro spray mist control. Agric. Res. Arid Areas 38, 159–167. doi: 10.7606/j.issn.1000-7601.2020.04.20

[B29] Luan X. Vico G. (2021). Canopy temperature and heat stress are increased by compound high air temperature and water stress and reduced by irrigation – a modeling analysis. Hydrol. Earth Syst. Sci. 25, 1411–1423. doi: 10.5194/hess-25-1411-2021

[B30] Luoyang Water Resources Bureau . (2022). Luoyang Water Resources Bulletin 2021. Luoyang: Luoyang Water Resources Bureau.

[B31] Nikolskii Y. Aidarov I. (2023). “ Over-irrigation and adverse effects,” in Handbook of Irrigation Hydrology and Management ( CRC Press, Boca Raton), 389–404. doi: 10.1201/9781003353928-26

[B32] Ning W. Zhang F. Zhang M. (2024). Trend prediction and influencing factors of the production comparative advantage of China’s main apple-producing provinces. PloS One 19, e0311912. doi: 10.1371/journal.pone.0311912 39423223 PMC11488726

[B33] Ou L. Zhang Y. Zhang Z. Chen Y. Wang K. Wen Y. . (2023). The relationship between canopy microclimate, fruit and seed yield, and quality in Xanthoceras sorbifolium. J. Plant Physiol. 284, 153975. doi: 10.1016/j.jplph.2023.153975 37028192

[B34] Pieper J. R. Anthony B. M. Chaparro J. M. Prenni J. E. Minas I. S. (2024). Rootstock vigor dictates the canopy light environment that regulates metabolite profile and internal fruit quality development in peach. Plant Physiol. Biochem. 208, 108449. doi: 10.1016/j.plaphy.2024.108449 38503188

[B35] Reta K. Netzer Y. Lazarovitch N. Fait A. (2025). Canopy management practices in warm environment vineyards to improve grape yield and quality in a changing climate. A review a vademecum to vine canopy management under the challenge of global warming. Sci. Hortic. 341, 113998. doi: 10.1016/j.scienta.2025.113998 38826717

[B36] Rodrigues M. Carvalho L. Gonçalves M. Ferreira S. de Sousa M. L. (2025). Sustainable strategies for sunburn mitigation in Gala apple orchards: Effects on yield, fruit quality, and plant physiology. Appl. Sci. 15, 11644. doi: 10.3390/app152111644 30654563

[B37] Saleem A. Anwar S. Nawaz T. Fahad S. Saud S. Ur Rahman T. . (2025). Securing a sustainable future: the climate change threat to agriculture, food security, and sustainable development goals. J. Umm Al-Qura Univ. Appl. Sci. 11, 595–611. doi: 10.1007/s43994-024-00177-3 30311153

[B38] Shi P. Yang J. Yang Y. Li Z. (2025). Connection between water consumption of apple production and subsurface water depletion on the Loess Plateau of China. Agric. Water Manage. 315, 109546. doi: 10.1016/j.agwat.2025.109546 38826717

[B39] Taherdoost H. Mohebi A. (2024). A comprehensive guide to the COPRAS method for multi-criteria decision making. J. Manage. Sci. Eng. Res. 7, 1–14. doi: 10.30564/jmser.v7i2.6280

[B40] Tong X. Wu P. Liu X. Zhang L. Zhou W. Wang Z. (2022). A global meta-analysis of fruit tree yield and water use efficiency under deficit irrigation. Agric. Water Manage. 260, 107321. doi: 10.1016/j.agwat.2021.107321 38826717

[B41] Veisi H. Deihimfard R. Shahmohammadi A. Hydarzadeh Y. (2022). Application of the analytic hierarchy process (AHP) in a multi-criteria selection of agricultural irrigation systems. Agric. Water Manage. 267, 107619. doi: 10.1016/j.agwat.2022.107619 38826717

[B42] Verma P. Bodh S. (2025). “ Introduction to growth and development of fruit crops,” in Advances in Growth Regulation of Fruit Crops ( CRC Press, Boca Raton), 1–10. doi: 10.1201/9781003354055-1

[B43] Wang H. Zhang S. Wang Z. Li D. Yan L. Feng Y. . (2024). Resistance index and browning mechanism of apple peel under high temperature stress. Hortic. Plant J. 10, 305–317. doi: 10.1016/j.hpj.2022.10.013 38826717

[B44] Weng J. Rehman A. Li P. Chang L. Zhang Y. Niu Q. (2022). Physiological and transcriptomic analysis reveals the responses and difference to high temperature and humidity stress in two melon genotypes. Int. J. Mol. Sci. 23, 734. doi: 10.3390/ijms23020734 35054918 PMC8776189

[B45] Wünsche J. N. Lombardini L. Greer D. H. (2004). SURROUND” particle film applications - effects on whole canopy physiology of apple. Acta Hortic. 636, 565–571. doi: 10.17660/ActaHortic.2004.636.72

[B46] Xu Q. Ma J. Chen R. Li X. Sun X. Zheng L. (2024). Variation of leaf turgor and pressure parameters evaluation in drip-irrigated apple canopy. Sci. Hortic. 331, 113188. doi: 10.1016/j.scienta.2024.113188 38826717

[B47] Xue R. Zhang C. Yan H. Disasa K. N. Lakhiar I. A. Akhlaq M. . (2025). Determination of the optimal frequency and duration of micro-spray patterns for high-temperature environment tomatoes based on the Fuzzy Borda model. Agric. Water Manage. 307, 109240. doi: 10.1016/j.agwat.2024.109240 38826717

[B48] Xue R. Zhang C. Yan H. Lakhiar I. A. Disasa K. N. Zhou Y. . (2024). Evaluating effect of micro-spray on tomatoes for resisting summer heat stress using a Fuzzy Borda combination evaluation model. Environ. Exp. Bot. 218, 105605. doi: 10.1016/j.envexpbot.2023.105605 38826717

[B49] Xue R. Zhang C. Yan H. Li J. Ren J. Akhlaq M. . (2023). Physiological response of tomato and cucumber plants to micro-spray in high-temperature environment: A scientific and effective means of alleviating crop heat stress. Agronomy 13, 2798. doi: 10.3390/agronomy13112798 30654563

[B50] Yang C. Tang Z. Wang G. Gessler A. Zhai B. Sun S. . (2025). Age‐dependent variations in xylem hydraulic efficiency and safety of Abies fabri. Physiol. Plant 177, e70271. doi: 10.1111/ppl.70271 40364791

[B51] Zhang H. Liu H. M. Yang X. Gong H. J. (2022). Effects of different micro-spray heights on the microclimate factors and quality under the grape trellis. Water Sav. Irrig., 97–100.

[B52] Zhang W. Lu J.-S. Bai J. Khan A. Zhao L. Wang W. . (2024). Reduced fertilization boosts soil quality and economic benefits in semi-arid apple orchard: A two-year appraisal of fertigation strategy. Agric. Water Manage. 295, 108766. doi: 10.1016/j.agwat.2024.108766 38826717

[B53] Zhang Y.-Q. Wen Y. Bai Q. Ma Z. Ye H.-L. Su S.-C. (2019). Spatio-temporal effects of canopy microclimate on fruit yield and quality of Sapindus mukorossi Gaertn. Sci. Hortic. 251, 136–149. doi: 10.1016/j.scienta.2019.02.074 38826717

[B54] Zhao B. Liu Q. Luo L. Zhou H. Zhang X. Ma F. . (2025). Suppression of MdPRP6 enhances adaptation of apple plants to long‐term drought. Physiol. Plant 177, e70099. doi: 10.1111/ppl.70099 39921481

[B55] Zhen J. Wang Y. Xia H. Li H. Wu H. Zhao C. . (2025). The relationship between microclimate factors and fruit quality in different tree canopies of Xiahui No. 8 peach trees. Front. Plant Sci. 16, 1551110. doi: 10.3389/fpls.2025.1551110 40538875 PMC12176857

[B56] Zheng M. Bai Y. Zhang J. Liu H. Cai J. Bai S. . (2022). Effects of shading and micro‐spray of canopy on photosynthetic characteristics, quality and yield of drip‐irrigated grapes ^*^. Irrig. Drain. 71, 23–34. doi: 10.1002/ird.2629 41531421

[B57] Zheng M. Bai Y. Zhang J. Liu H. Ding P. (2021). Effect of the mist micro-spray time on photosynthetic spatial heterogeneity of grape canopy. Eng. Agríc. 41, 39–46. doi: 10.1590/1809-4430-eng.agric.v41n1p39-46/2021 41099703

[B58] Zheng M. Sun Y. Mu W. Bai Y. Wang Q. Lu Z. . (2025). Effects of subsurface drip irrigation depth on growth characteristics and yield quality of apples (Malus pumila Mill.) in Northwest China. Plants 14, 2702. doi: 10.3390/plants14172702 40941867 PMC12430693

[B59] Zhong Y. Fei L. Li Y. Zeng J. Dai Z. (2019). Response of fruit yield, fruit quality, and water use efficiency to water deficits for apple trees under surge-root irrigation in the Loess Plateau of China. Agric. Water Manage. 222, 221–230. doi: 10.1016/j.agwat.2019.05.035 38826717

[B60] Zhou H. Ma L. Zhang S. Zhao L. Niu X. Qin L. . (2023). Effect of water-fertilizer coupling on the growth and physiological characteristics of young apple trees. Agronomy 13, 2506. doi: 10.3390/agronomy13102506 30654563

